# Molecular basis for CPAP-tubulin interaction in controlling centriolar and ciliary length

**DOI:** 10.1038/ncomms11874

**Published:** 2016-06-16

**Authors:** Xiangdong Zheng, Anand Ramani, Komal Soni, Marco Gottardo, Shuangping Zheng, Li Ming Gooi, Wenjing Li, Shan Feng, Aruljothi Mariappan, Arpit Wason, Per Widlund, Andrei Pozniakovsky, Ina Poser, Haiteng Deng, Guangshuo Ou, Maria Riparbelli, Callaini Giuliano, Anthony A. Hyman, Michael Sattler, Jay Gopalakrishnan, Haitao Li

**Affiliations:** 1 Beijing Advanced Innovation Center for Structural Biology, Department of Basic Medical Sciences, School of Medicine, Tsinghua University, Beijing 100084, China; 2 MOE Key Laboratory of Protein Sciences, School of Life Sciences, Tsinghua University, Beijing 100084, China; 3 Tsinghua-Peking Center for Life Sciences, Tsinghua University, Beijing 100084, China; 4 Institute for Biochemistry I and Center for Molecular Medicine of the University of Cologne, Robert-Koch-Str. 21, Cologne 50931, Germany; 5 Institute of Structural Biology, Helmholtz Zentrum München, Ingolstädter Landstr. 1, Neuherberg 85764, Germany; 6 Biomolecular NMR at Center for Integrated Protein Science Munich and Department Chemie, Technische Universität München, Lichtenbergstr. 4, Garching 85747, Germany; 7 Department of Life Sciences, University of Siena, Siena 53100, Italy; 8 Max Planck Institute of Molecular Cell Biology and Genetics, Pfotenhauer Str. 108, Dresden 01307, Germany; 9 Collaborative Innovation Center for Biotherapy, West China Hospital, Sichuan University, Chengdu 610041, China

## Abstract

Centrioles and cilia are microtubule-based structures, whose precise formation requires controlled cytoplasmic tubulin incorporation. How cytoplasmic tubulin is recognized for centriolar/ciliary-microtubule construction remains poorly understood. Centrosomal-P4.1-associated-protein (CPAP) binds tubulin via its PN2-3 domain. Here, we show that a C-terminal loop-helix in PN2-3 targets β-tubulin at the microtubule outer surface, while an N-terminal helical motif caps microtubule's α-β surface of β-tubulin. Through this, PN2-3 forms a high-affinity complex with GTP-tubulin, crucial for defining numbers and lengths of centriolar/ciliary-microtubules. Surprisingly, two distinct mutations in PN2-3 exhibit opposite effects on centriolar/ciliary-microtubule lengths. CPAP^F375A^, with strongly reduced tubulin interaction, causes shorter centrioles and cilia exhibiting doublet- instead of triplet-microtubules. CPAP^EE343RR^ that unmasks the β-tubulin polymerization surface displays slightly reduced tubulin-binding affinity inducing over-elongation of newly forming centriolar/ciliary-microtubules by enhanced dynamic release of its bound tubulin. Thus CPAP regulates delivery of its bound-tubulin to define the size of microtubule-based cellular structures using a ‘clutch-like' mechanism.

## Abstract (web summary)

Centrioles and cilia are microtubule-based structures of defined architecture, but what regulates this architecture is not clear. Here the authors discover that centrosomal-P4.1-associated-protein (CPAP) binds the α/β-tubulin dimer and licenses it for tubulin delivery contributing to centriolar/ciliary length and architecture control.

## Graphical abstract

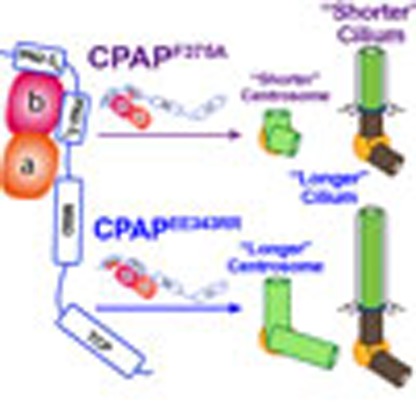

Centrioles are microtubule-based eukaryotic structures that build centrosomes and cilia, which are required for accurate cell division and cellular signaling^1^,^2^,^3^,^4^,^5^. Centrioles have highly conserved architecture displaying defined numbers and lengths of microtubules. Tubulin heterodimers (hereafter tubulin) are the building blocks of centriolar- and ciliary-microtubules. During cilium construction, intraflagellar transport (IFT) machineries mediate the transport of ciliary building blocks of tubulin from the cytoplasmic ciliary base to the tip^6^,^7^,^8^. However, it remains poorly understood how a fraction of tubulin is selected from its large cytoplasmic pool and the mechanisms that operate to deliver these specialized tubulin to construct defined lengths of centriolar- and ciliary-microtubules.

Since cilium templates from a centriole that resides within a centrosome, it is conceivable that a centrosomal protein that can directly interact with cytoplasmic tubulin could play a role in selective regulation of tubulin incorporation during centriolar- and ciliary-microtubule construction. Among the centrosomal proteins, CPAP and its ortholog Sas-4 plays roles in centriolar- and ciliary-microtubule elongation^9^,^10^,^11^,^12^,^13^,^14^. Data have shown that these functions require CPAP-tubulin interaction. Specifically, a CPAP/Sas-4 mutant that does not bind tubulin caused shortening of centrioles and primary cilia^9^,^10^,^11^. These data suggest the possibility that CPAP could play a role in delivering its bound tubulin at the site of centriole assembly and/or building centriolar-microtubules.

Free tubulin is ubiquitously present in high amounts in cells in contrast to CPAP, which has lower expression levels^9^,^15^,^16^. It is known that CPAP *via* its conserved PN2-3 domain (amino acids 319–394) sequesters free tubulin into a non-polymerizable 1:1 complex^17^,^18^. Thus at equilibrium, the amount of CPAP bound cytoplasmic tubulin that is unavailable for polymerization would only be a small fraction of the free tubulin. This suggests that the small fraction of CPAP-bound tubulin is required in certain CPAP-specific processes that are regulated in a spatiotemporal manner such as centriolar- and ciliary-microtubule size control. While recent studies have substantially improved our understanding on the significance of CPAP-tubulin interaction in centriole biogenesis and pericentriolar material recruitment, its significance in centriolar- and ciliary-microtubule construction remains unclear^9^,^11^,^12^,^13^,^14^. Thus, understanding the molecular basis of PN2-3-tubulin interaction is crucial in dissecting how cytoplasmic tubulin is sequestered by CPAP for the controlled delivery to define centriolar- and ciliary-microtubule lengths in cells.

In this study, we therefore investigated the structural basis of CPAP-tubulin interaction and identified that CPAP via its conserved PN2-3 domain forms a high affinity complex with GTP-tubulin and prevents it from polymerization. Our functional studies in human cells and flies then identified that, through the different facets of its PN2-3 domain, CPAP defines centriolar- and ciliary-microtubule lengths, firstly, through the sufficient binding of cytoplasmic tubulin and secondly by regulated release of its bound tubulin to define centriolar- and ciliary-microtubule lengths.

## Results

### Molecular basis for PN2-3-tubulin interaction

**Figure 1:**
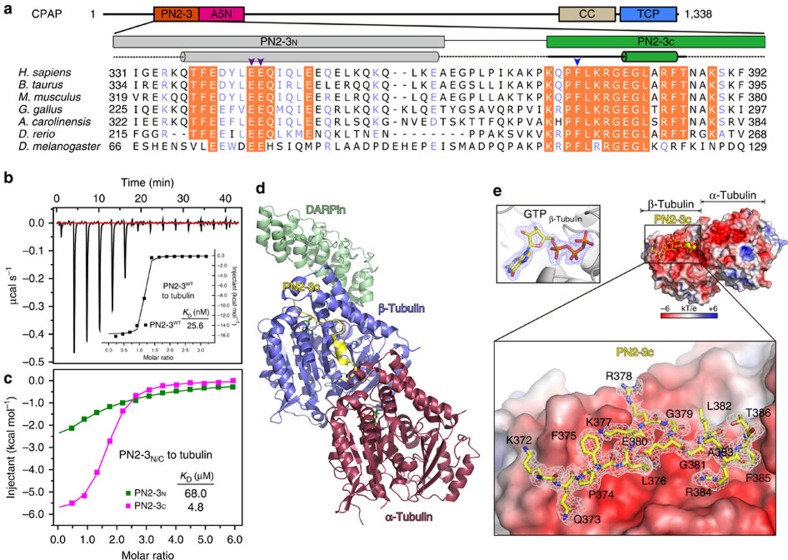
Molecular basis for PN2-3-tubulin interaction. (**a**) Domain architecture of CPAP and sequence conservation analysis of PN2-3_N_ and PN2-3_C_. Arrowheads mark E343-E344 (purple) and F375 (blue). (**b**) ITC titration and fitting curve of wild type (WT) PN2-3 with tubulin. (**c**) Overlay of ITC fitting curves of PN2-3_N_ (green) and PN2-3_C_ (magenta) titrated into tubulin. (**d**) Ribbon view of PN2-3_C_-GTP-tubulin-DARPin complex. Yellow dot line indicates the invisible region of PN2-3_C_ in density map. GTP, green sticks. (**e**) Close-up view of PN2-3_C_ loop-helix motif (yellow) docked onto β-tubulin outer surface colored by electrostatic potential (−6 kT e^−1^, red; +6 kT e^−1^, blue). Fo-Fc omit map (1.5 σ) of PN2-3_C_ was shown as grey meshes. Top-left, Fo-Fc omit map (blue meshes, 4.5 σ) of GTP.

**Figure 2:**
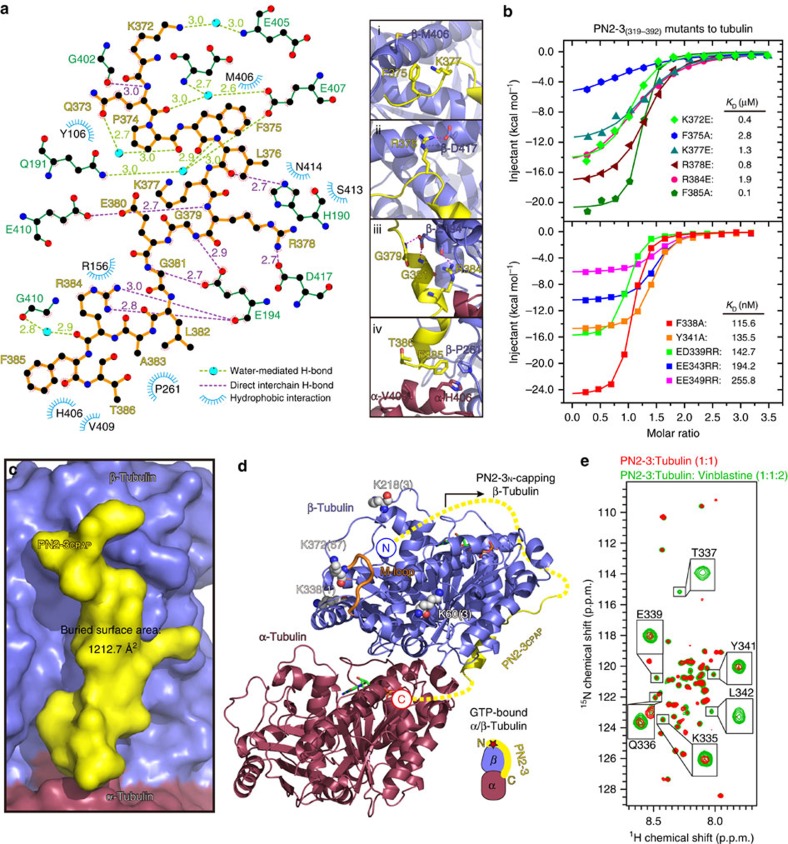
Interaction details of PN2-3_C_-tubulin and recognition mode for PN2-3_N._ (**a**) Left, LigPlot diagram of PN2-3_C_ and tubulin binding details. The PN2-3_C_ was colored dark yellow and depicted as thick lines. Right, (i–iv) close-up view and structural illustration of key interaction pairs listed in the LigPlot diagram. Overall structures are in ribbon view and key residues are in stick view. Yellow, PN2-3_C_; raspberry, α-tubulin; slate, β-tubulin. (**b**) ITC fitting curves of mutant PN2-3 titrated into tubulin dimer with the upper figure for mutations in PN2-3_C_ and the lower figure for mutations in PN2-3_N_. (**c**) Surface view of PN2-3_C_ bound to a tubulin dimer. PN2-3_C_, β-tubulin, α-tubulin are colored yellow, slate, and raspberry, respectively. Note, PN2-3_C_ binds to the microtubule outer surface in an anti-parallel orientation along the longitudinal axis of microtubule. (**d**) Structural annotation of CL-MS results over a tubulin dimer. White spheres, lysine residues that cross-link with PN2-3N-terminal amine. Parenthesis, number of CL-MS peaks detected. (**e**) Overlay of ^1^H,^15^N-SOFAST HMQC spectra of tubulin-free PN2-3 (black, Supplementary Fig. 4) and tubulin-bound PN2-3 (red) at equimolar ratios. Disappearance/broadening of PN2-3 peaks indicate its binding to tubulin. Upon addition of 2-fold excess of vinblastine (green), some PN2-3 peaks reappear, indicating that vinblastine interferes with PN2-3-tubulin interaction.

The PN2-3 domain, at the N-terminus of CPAP, is conserved from invertebrates to vertebrates (Fig. 1a). Using isothermal titration calorimetry (ITC), we determined a 25.6 nM binding affinity between PN2-3 and tubulin (Fig. 1b), and found PN2-3_C_ (PN2-3's C-terminus, aa 372-394) is sufficient to bind tubulin (Fig. 1c). By adopting a DARPin strategy, in which DARPin molecule didn't affect binding of PN2-3_C_ to tubulin, we solved the 2.1 Å crystal structure of bovine tubulin bound to PN2-3_C_ (ref. 19) (Fig. 1d,e, Table 1 and Supplementary Fig. 1). In this structure, a loop-helix motif spanning residues 372–386 of PN2-3_C_ targets an acidic microtubule outer surface on β-tubulin, although the last eight C-terminal residues (aa 387–394) were not traceable in the density map (Fig. 1e). A GTP molecule is identified in the β-tubulin nucleotide-binding pocket despite tubulin exhibiting a curved conformation (Fig. 1e and Supplementary Fig. 2). Recognition specificity arises from a number of water-mediated and direct interchain hydrogen bonds, and hydrophobic contacts between β-tubulin and PN2-3_C_ (Fig. 2a). Key interacting residues within PN2-3_C_ were then confirmed by structure-guided mutagenesis and ITC (Fig. 2b). Notably, F375A caused a drastic 109-fold drop in affinity from 25.6 nM to 2.8 μM, indicating its significance in forming a high-affinity CPAP-tubulin complex. We observed a perfect shape complementarity between the crouched PN2-3_C_ loop-helix and its binding groove along the vertical axis for tubulin polymerization (Fig. 2c).

The N-terminal helical region of PN2-3 (PN2-3_N_, aa 323–361) alone has weak tubulin binding affinity (*K*_D_=68.0 μM) (Fig. 1c). However, together with PN2-3_C_, it contributes a ∼140-fold increased binding affinity (*K*_D_=25.6 nM) (Fig. 1b). As the PN2-3_N_-tubulin co-crystal structure could not be resolved, we used cross-linking mass spectrometry (CL-MS) to identify PN2-3_N_ contacts with tubulin (Fig. 2d and Supplementary Fig. 3). CL-MS analysis indicates that PN2-3_N_ wraps around the microtubule α-β-tubulin interface and makes contacts at the β-tubulin's microtubule-lumen surface, as a majority of cross-linking peaks were identified between PN2-3-N-terminal amine and lysine 372 in proximity to the β-tubulin M-loop (Fig. 2d). Such a binding mode is further supported by competition nuclear magnetic resonance spectroscopy (NMR) titration, where the weakly binding PN2-3_N_ region can be displaced by the high affinity ligand vinblastine, which is known to bind to the microtubule α-β-tubulin interface, but not by colchicine, which binds to the β-α interface within a tubulin dimer (Fig. 2e and Supplementary Fig. 4). Arginine substitutions of the conserved PN2-3_N_'s E343 and E344 (hereafter EE343RR) caused a 8-fold reduced interaction (*K*_D_=194.2 nM) (Fig. 2b).

We then compared the NMR spectra of wild type and mutant (PN2-3^F375A^ and PN2-3^EE343RR^) PN2-3 fragments, which exhibited numerous chemical shift differences between PN2-3^WT^ and PN2-3^EE343RR^. This suggests a potential disruption of the helix, which extends from residues F338-E368 (Supplementary Fig. 4c). On the other hand, we noticed similar spectra of PN2-3^WT^ and the PN2-3^F375A^, which is further confirmed by the comparison of PN2-3^F375A^ and PN2-3^EE343RR^ spectra. Upon addition of unlabeled tubulin at equimolar ratio to ^15^N-labeled PN2-3^WT^, PN2-3^F375A^ and PN2-3^EE343RR^ mutants, we observed line broadening/disappearance of the peaks, which is suggestive of binding. Exchange broadening can become obvious in low affinity (μM *K*_D_) binding events. Accordingly, we observed that line broadening increases from PN2-3^WT^ to PN2-3^EE343RR^ and is the highest with the PN2-3^F375A^ mutant, as the affinity for tubulin decreases in the same order agreeing with our ITC titrations (Figs 1b and 2b).

Together, our structural and mutagenesis data indicate that PN2-3 wraps around β-tubulin like a necklace with its PN2-3_N_ covering the microtubule α-β and lumen surfaces of β-tubulin and its PN2-3_C_ occluding the β-tubulin microtubule outer surface (Fig. 2c,d and Supplementary Fig. 3d). This unique binding mode suggests that PN2-3 could prevent the polymerization of its bound tubulin (Supplementary Fig. 3e).

### PN2-3 prevents its bound tubulin from polymerization

To test if PN2-3 could bind tubulin and prevent the bound tubulin from polymerization, we performed microtubule-pelleting assays using free tubulin and PN2-3 variants (Supplementary Fig. 5a). Tubulin at a high concentration self-polymerizes into microtubules. While polymerized tubulin could be pelleted by centrifugation, free tubulin remains in solution in the supernatant^17^. When we included PN2-3 variants in this assay, we found that in contrast to PN2-3^WT^, PN2-3^F375A^ did not prevent tubulin polymerization (Supplementary Fig. 5b). This could be due to the presence of F375, which is required for the formation of high affinity CPAP-tubulin complex (Fig. 2b). In addition, F375-containing PN2-3_C_ domain binds the β-tubulin at the microtubule outer surface, which might allosterically disrupt microtubule formation by inducing or stabilizing a curved tubulin dimer (Supplementary Fig. 2). Supporting this notion, PN2-3_C_ alone was sufficient to prevent tubulin from polymerization (Supplementary Fig. 5c). PN2-3_N_ caps β-tubulin and conceivably has intrinsic microtubule depolymerization activities. However, compared to PN2-3_C_, PN2-3_N_ is not sufficient to prevent microtubule polymerization (Supplementary Fig. 5c). This is likely due to the weak binding affinity observed for PN2-3_N_ and tubulin (*K*_D_=68 μM) as compared to the case of PN2-3_C_ (*K*_D_=4.8 μM) (Fig. 1c). Similarly, PN2-3_C_^F375A^ failed to prevent tubulin from polymerization due to loss of PN2-3-tubulin binding (Supplementary Figs 5c and 6a). We then tested the effects of PN2-3 variants in cytoplasmic microtubules by overexpressing green fluorescent protein (GFP)-tagged PN2-3 variants in HeLa cells. In contrast to PN2-3^WT^, overexpression of PN2-3^F375A^ did not cause the collapse of cytoplasmic microtubules indicating that *in vitro,* the ability of PN2-3 to bind tubulin and prevent it from polymerization is F375-dependent (Supplementary Fig. 6b).

### CPAP-tubulin regulates centriole and cilium lengths

First, we tested the functional significance of CPAP's F375-tubulin interaction in centrosome biogenesis. To test this, we established stable HeLa lines expressing RNAi-resistant GFP-tagged CPAP, and CPAP^F375A^ using bacterial artificial chromosome recombineering (BACs). This system allows expression of gene products under their own endogenous promoters^20^,^21^. As described previously, treating cells with CPAP-specific siRNA depleted endogenous protein, retaining the RNAi-resistant CPAP (Supplementary Fig. 7a). Expressing wild type siRNA-resistant CPAP completely rescued the effects of siRNA-mediated CPAP depletion as cells proliferated without centrosome duplication defects. In contrast, RNAi-resistant CPAP^F375A^ failed to rescue the centrosome duplication phenotype caused by wild type CPAP depletion. Specifically, when cultured for prolonged periods of time, we noticed a proportion of cells displaying less than two centrosomes reaffirming previous findings that CPAP-tubulin interaction is required for centrosome duplication in cultured human cells^11,12^ (Supplementary Fig. 7b)

**Figure 3:**
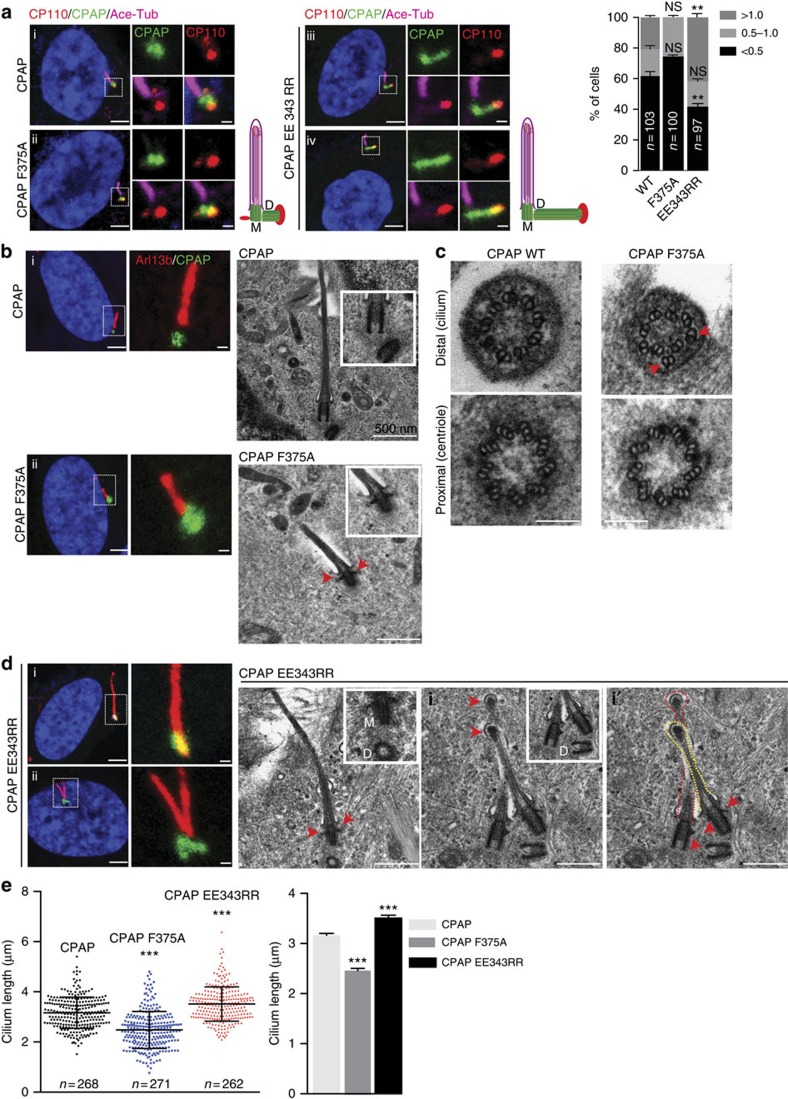
Effects of CPAP-tubulin interaction on centriolar- and ciliary-microtubule formation in human cells. CPAP^EE343RR^ expression but not CPAP or CPAP^F375A^ (green) causes overly long daughter centriolar- (**a**) and ciliary-microtubules (**b**). The centrosomal protein CP110 (red) caps the distal end of daughter centrioles in (**a**), which does not template cilium formation. Schematic is given at right. Acetylated-α-tubulin (magenta) in (**a**) or Arl13b (red) in (**d**) labels cilia. A bar diagram at top right quantifies percentage of cells and their centriolar lengths (*n*=103 for WT, *n*=100 for CPAP^F375A^ and *n*=97 for CPAP^EE343RR^), ANOVA, **P<0.001. Error bars represent±s.e.m. Number of experiments (N)=3. Note that the exact lengths of CPAP^F375A,^ centrioles could sometimes not be measured due to their small size. EM-micrographs of cilia in cells expressing the respective CPAP variants are given at the sides of light microscopy images. Arrows mark centriolar-appendages. (**c**) Serial section-EM of centriole and cilium from cells expressing CPAP^WT^ or CPAP^F375A^. Arrows mark the abnormally organized ciliary microtubule doublets (*n*=32). Scale bar 100 nm. (**d**) CPAP^EE343RR^ expression causes biciliation (i and i'). Serial sectioning-EM showing single- and double-cilia (arrowheads and red/yellow dotted lines). Red arrows mark centriolar-appendages. In biciliated cells, mother and/or matured daughter centrioles template the formation of cilia. M, mother centriole. D, daughter centriole. Scale bar 500 nm. (**e**) Ciliary-length quantifications. ANOVA, ***P<0.0001, *n*>250. The error bars represent±s.e.m. Number of experiments (N)=3. Scale bar 1 μm.

Since, depleting endogenous CPAP perturbs centrosome duplication, we took an alternative approach to study the role of CPAP-tubulin interaction in regulating centriolar- and ciliary-microtubule lengths. We overexpressed GFP-tagged CPAP^WT^ and CPAP^F375A^ in RPE1 cells using lentiviral-transduction. We analyzed ciliated cells and determined daughter centrioles from mother centrioles by immunostaining CP110, a centrosomal protein that localizes to the distal end of daughter centrioles providing a cap-like structure^14^,^22^. CP110 predominantly localizes to the daughter centriole, which does not template the formation of cilium (Fig. 3a). Compared to CPAP^WT^ expression, CPAP^F375A^, which fails to efficiently bind tubulin, resulted in shorter centrioles. These data suggest the need of a high-affinity CPAP-tubulin interaction for centriolar-microtubule elongation (Fig. 3ai-ii).

We then expressed CPAP^EE343RR^ that perturbs PN2-3's N-terminal helix, which caps the microtubule lumen surface of β-tubulin and possibly unmasks the β-tubulin surface for polymerization (Fig. 2d)^23^,^24^. To our surprise, CPAP^EE343RR^ expression caused overly long mother and daughter centrioles. In contrast to most of the CPAP^F375A^ centrioles, which were shorter in length, we could readily distinguish and measure the lengthy centrioles induced by CPAP^EE343RR^. The finding that CPAP^EE343RR^ causes longer daughter centrioles, suggests that CPAP^EE343RR^ can affect newly forming microtubule-based structures (Fig. 3aiii-iv and Supplementary Fig. 8b).

Next, we analyzed the significance of CPAP-tubulin interaction in ciliogenesis. Although CPAP^F375A^ expressing cells could assemble cilia, they were shorter in length. Serial sectioning-electron microscopy (EM) identified that they appear structurally normal until the transition zone where they displayed abnormal axonemes with distorted doublet-microtubules (Fig. 3b,c,e and Supplementary Fig. 9). On the other hand, expressing CPAP^EE343RR^ resulted in structurally normal but overly long cilia also causing ∼10% of cells to be biciliated (Fig. 3d,e, Supplementary Figs 9 and 10a). EM analyses revealed that biciliated cells contained four centrioles, in which two of them template cilium formation consistently displaying centriolar-appendages, indicating that they are either the mother and/or matured daughter centriole. This data suggests that CPAP^EE343RR^ not only favors centriolar-microtubule elongation but also promotes daughter centrioles to form cilia (Fig. 3d,e and Supplementary Figs 9 and 10b,c).

To exclude that overly long cilia in cells expressing CPAP^EE343RR^ is due to defective ciliary disassembly, we synchronized RPE-1 cells expressing doxycycline inducible CPAP^EE343RR^ at G_0_ by serum starvation for 96 h (ref. 25). The doxycycline inducible system prevented cells from forming long cilia caused by constitutive expression of CPAP^EE343RR^. Upon serum stimulation and doxycycline induction, the percentages of cells expressing CPAP^WT^ and CPAP^EE343RR^ re-entering cell cycle concurrent with cilium disassembly did not differ. This excluded the involvement of defective ciliary disassembly in causing long cilia (Supplementary Fig. 11).

### CPAP via PN2-3 controls dynamic release of its bound tubulin

Based on the above findings (Figs 1, 2, 3), we speculated that in the cytoplasm, CPAP binds GTP-tubulin forming a high-affinity cytoplasmic complex and prevents the bound-tubulin from spontaneous polymerization until targeted to centriolar- and ciliary-microtubule elongation sites during the early phase of centriole/cilium formation. The experiment that supports this idea is the finding of shorter centriolar- and ciliary-microtubules upon CPAP^F375A^ expression, a variant with strongly reduced tubulin interaction (Fig. 3a). At the site of centriolar- and ciliary-microtubule elongation, a mechanism that operates to control tubulin release could then regulate microtubule growth. One factor that could contribute to this regulation to facilitate tubulin release is the uncapping of CPAP's PN2-3_N_ from β-tubulin (Supplementary Fig. 12a). It is also known that CPAP/Sas-4 is a dynamically regulated cell cycle protein with cytoplasmic and centriolar pools^11^,^15^. Thus, centriolar CPAP could be an atypical centriolar-microtubule builder, regulating microtubule growth to define centriolar lengths. This is demonstrated by the overly long centrioles observed upon CPAP^EE343RR^ expression, a variant with reduced tubulin affinity and unmasking β-tubulin surface making it available for tubulin polymerization (Fig. 3 and Supplementary Fig. 12). If this were true, CPAP^EE343RR^ would exhibit an enhanced release of its bound-tubulin via a ‘clutch-like' mechanism so as to favor microtubule growth at the onset of centriolar-microtubule assembly (Supplementary Fig. 12a).

**Figure 4:**
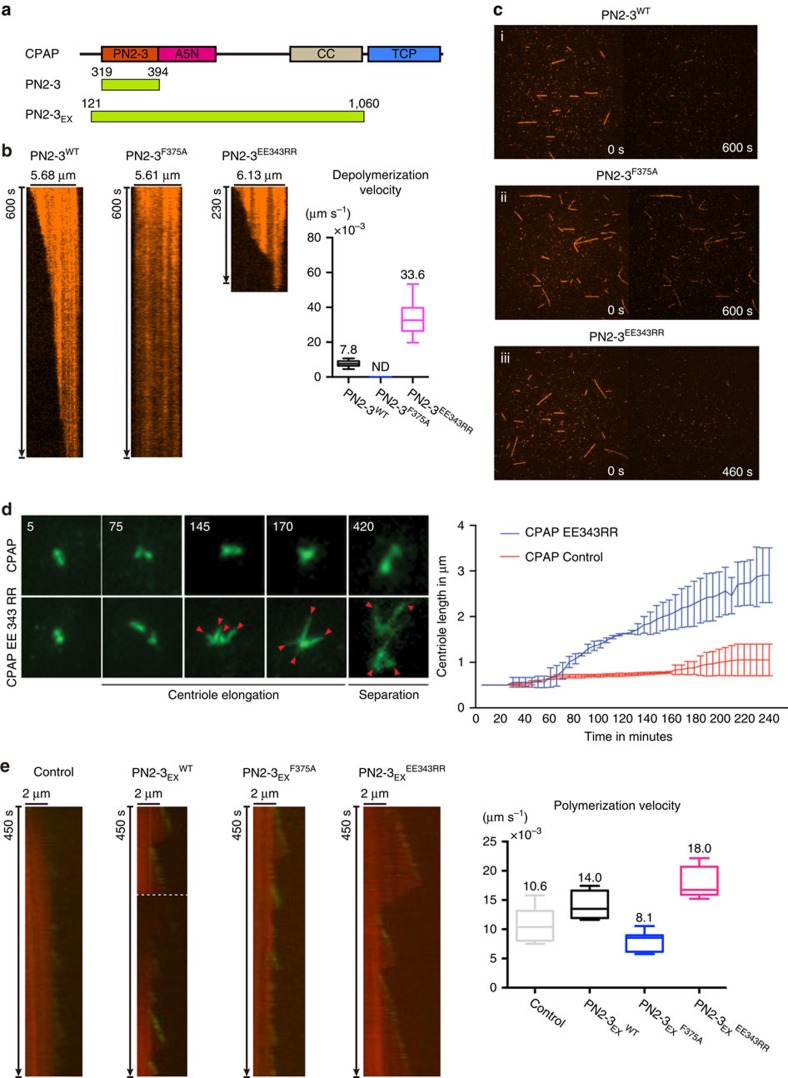
Microtubule end-tracking and live-imaging measurements of centriolar growth rates. (**a**) Schematic view of PN2-3 and PN2-3_EX_ constructs. (**b**) Kymographs and averaged depolymerization velocities of PN2-3^WT^, PN2-3^F375A^ and PN2-3^EE343RR^ during microtubule end-tracking assay. (**c**) Snapshots of microtubule end-tracking assays of PN2-3^WT^ (i), PN2-3^F375A^ (ii), and PN2-3^EE343RR^ (iii) at different time points. (**d**) Expression of CPAP^EE343RR^-GFP but not CPAP^WT^-GFP causes a rapid centriolar-elongation that is distinct enough to measure in contrast to CPAP^WT^ expression. Arrowheads mark elongating (at 145th and 170th mins) and separating (at 420th mins) centrioles. Centriolar-growth rate curve is given (*n*=57, CPAP^EE343RR^; 50, CPAP^WT^). The error bars represent±s.d. Number of experiments (N)=3. (**e**) Kymographs and averaged polymerization velocities of PN2-3_EX_^WT^, PN2-3_EX_^F375A^, and PN2-3_EX_^EE343RR^ during microtubule polymerization experiments.

To test PN2-3's bound-tubulin releasing ability *in vitro*, we performed microtubule-end tracking assays using GMPCPP (a non-hydrolysable GTP analog) stabilized microtubules and PN2-3 variants (Fig. 4a–c). PN2-3, which can bind tubulin dimers at high affinity but not microtubules, has a microtubule-destabilizing activity primarily harbored in PN2-3_C_ (Supplementary Fig. 5)^17^,^18^. Thus, we measured the rate of PN2-3-mediated microtubule-destabilization as a function of its bound-tubulin releasing ability, with the assumption that PN2-3 is dynamically recycled for continuous microtubule disruption. Consistently, adding PN2-3^WT^ destabilized microtubules at a slower rate compared to PN2-3^EE343RR^, implying a faster rate of bound-tubulin release (Fig. 4b,c and Supplementary Movies 1–3). Importantly, these *in vitro* microtubule destabilization activities do not reflect how full-length CPAP functions in cells to regulate centriolar- and ciliary-microtubule growth with excess GTP-tubulin.

To directly analyze CPAP in elongating centrioles of living cells, we expressed inducible CPAP^WT^ and CPAP^EE343RR^ in RPE1 cells. Real time measurements reveal CPAP^EE343RR^ to have faster centriole elongation rates of ∼0.014 μm min^−1^ (CPAP^WT^=0.0017 μm min^−1^) reaching up to 3.7 μm in centriolar length. This suggests that CPAP regulates microtubule growth for defined centriolar-microtubule lengths (Fig. 4c, Supplementary Fig. 12b and Supplementary Movies 4–7). Consistently, our fixed experiments also revealed that CPAP^EE343RR^ caused a faster rate of ciliary-microtubule growth (Supplementary Fig. 12c). It thus appears that CPAP regulates the growth of microtubule-based structures through controlled release of its bound tubulin and its ability to function as a centriolar-microtubule builder, both of which occur at the site of centriole assembly.

### Extended PN2-3 promotes microtubule polymerization *in vitro*

In order to prove the microtubule polymerization activity of CPAP *in vitro*, we recombinantly prepared an extended CPAP frame spanning from PN2-3 to a C-terminal coiled coil (CC) motif (aa 121–1060, PN2-3_EX_; Fig. 4a) using mammalian cell expression system and conducted microtubule polymerization assays (Fig. 4e and Supplementary Movies 8–11). In an optimized microtubule growth condition in the presence of 15 μM GTP-tubulin, we could observe background microtubule growth at an average rate of ∼0.0106 μm s^−1^ without PN2-3_EX_. After adding 1 μM wild type PN2-3_EX_, the microtubule growth rate was increased to ∼0.014 μm s^−1^, suggesting a role of PN2-3_EX_ in prompting microtubule polymerization. Interestingly, PN2-3_EX_^F375A^ caused slight inhibition of microtubule polymerization with a growth rate of ∼0.0081 μm s^−1^. In consistence with our live imaging experiments of CAPA^EE343RR^, PN2-3_EX_^EE343RR^ displayed an enhanced microtubule growth with a rate of ∼0.018 μm s^−1^. In addition, GFP-tagged EB1 (refs 26, 27) was used to trace the plus end of growing microtubule. We observed active and dynamic plus end microtubule growth in the presence of PN2-3_EX_, especially in its EE343RR mutant form. These data suggest wild type and EE343RR mutant PN2-3_EX_ may localize at the plus end during the process of microtubule polymerization (Fig. 4e and Supplementary Movies 8–11). PN2-3_EX_ contains a tubulin-binding PN2-3 motif^28^, a microtubule-binding A5N motif^17^ and a dimerization CC motif^29^ (Fig. 4a), which likely constitute as a key element for the observed microtubule polymerization activity. In summary, our *in vitro* microtubule growth studies support a critical role of CPAP in regulating centriolar and ciliary length.

### Sas-4-tubulin controls centriole and cilium lengths *in vivo*

We then confirmed whether the Sas-4-tubulin interaction (the fly counterpart of CPAP) has a conserved function in controlling centriolar and ciliary length *in vivo.* To do this, we generated transgenic *Drosophila* expressing endogenous levels of GFP-tagged Sas-4^WT^, Sas-4^F112A^ (corresponding to CPAP^F375A^) and Sas-4^EE78RR^ (corresponding to CPAP^EE343RR^) in Sas-4 null flies (Supplementary Fig. 12d). In contrast to Sas-4^WT^ and Sas-4^EE78RR^, Sas-4^F112A^ failed to rescue the uncoordination and sterile phenotypes of null flies indicating that Sas-4^F112^-tubulin interaction is required to form functional sensory cilia and sperm flagella^9^,^30^.

**Figure 5:**
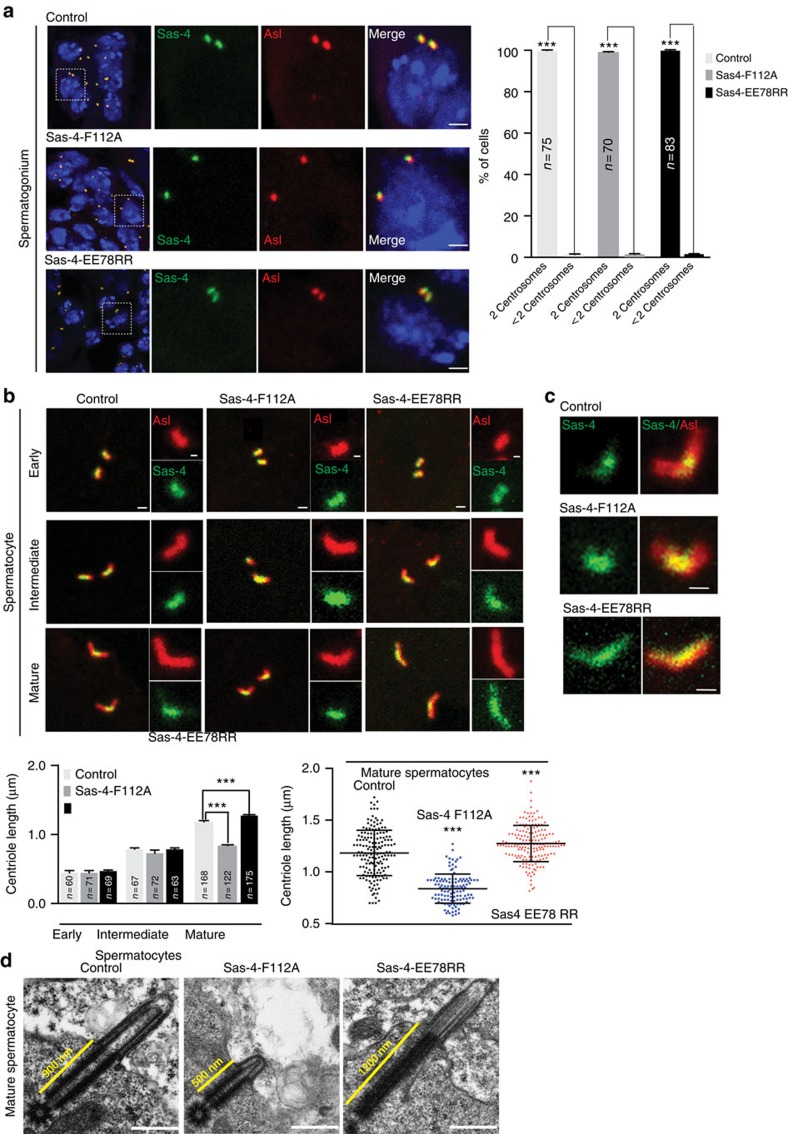
Effects of Sas-4-tubulin interaction on centriolar- and ciliary-microtubule formation in *Drosophila.* (**a**) Sas-4-tubulin interaction is dispensable for centrosome duplication in spermatogonium cells. Neither Sas-4 variant prevents centrosome duplication as numerically normal numbers of centrosomes were found in *Drosophila* expressing GFP tagged Sas-4^WT^, Sas-4^F112A^, or Sas-4^EE78RR^ in Sas-4 null flies. Quantification is shown at top right. Centrosomes are co-labeled with Asl (red) (*n*≥70), ANOVA, ***P<0.0001. Error bars represent±s.e.m. Number of experiments (N)=3. Scale bar, 1 μm. (**b**) Mature but not early or intermediate spermatocyte centrioles of *Drosophila* expressing Sas-4^EE78RR^ (green) display long centrioles. Asl (red) labels the elongating centrioles. Scale bar 1 μm (insets 0.5 μm). (**c**) Sas-4 localization in mature spermatocyte centrioles. In contrast to Sas-4^WT^ (control) and Sas-4^F112A^, Sas-4^EE78RR^ displays an extended localization all along the centriole length. Length measurements are given at bottom. ANOVA, ***P<0.0001, (*n*>120). The error bars represent±s.e.m. Scale bar 1 μm. (**d**) Longitudinal EM-sections of mature spermatocytes expressing Sas-4^WT^ (control), Sas-4^F112A^ and Sas-4^EE78RR^. Sas-4^EE78RR^ expression causes long centrioles. In contrast, Sas-4^F112A^ fails to elongate centrioles. Scale bar 500 nm.

However, Sas-4^F112A^ flies displayed correct centrosome numbers in spermatogonia indicating that Sas-4-tubulin interaction in flies is dispensable for centrosome duplication (Fig. 5a). This allowed us to specifically study the significance of Sas-4-tubulin interaction in centriole length determination *in vivo*. Analyzing mature spermatocytes of Sas-4^F112A^ flies, we found centrioles with significantly reduced lengths. On the other hand, Sas-4^EE78RR^ flies displayed overly elongated centrioles, consistent with observations in human cells (Fig. 5b,d). Examining Sas-4's localization at the elongating centrioles of spermatocytes, we consistently found a concentrated proximal localization of Sas-4^WT^. In contrast, Sas-4^EE78RR^ that causes elongated centrioles displayed an extended localization along the length of its overly elongated centrioles, coherent with our *in vitro* experiments suggesting that CPAP binds at the plus end of microtubule during polymerization (Figs 4e and 5b,c).

**Figure 6:**
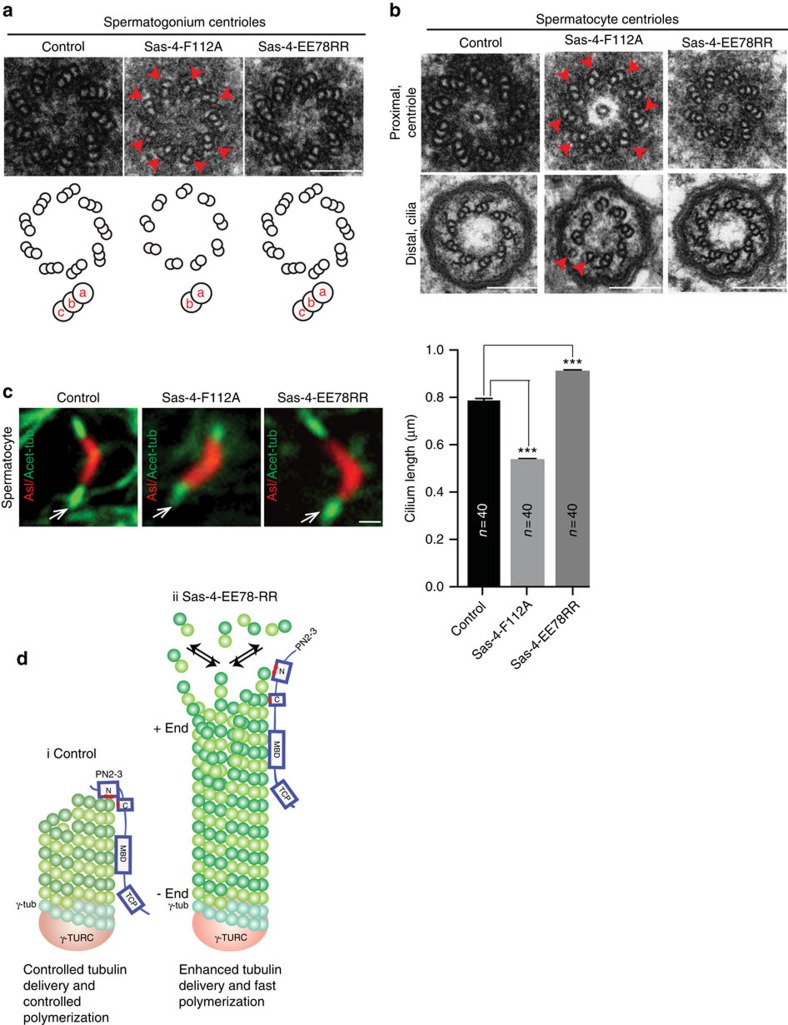
Effects of Sas-4-tubulin interaction on centriolar and ciliary architecture *in vivo*. EM-micrographs displaying centrioles of spermatogonium. Scale bar 100 nm. (**a**) and matured spermatocyte cells (**b**). In contrast to Sas-4^WT^ and Sas-4^EE78RR^, Sas-4^F112A^ centrioles contain doublet- instead of triplet-microtubules and aberrant ciliary microtubules (arrowheads and cartoon). (**c**) Primary cilia of matured spermatocytes expressing Sas-4^WT^, Sas-4^F112A^ and Sas-4^EE78RR^. Compared to Sas-4^WT^, Sas-4^EE78RR^ spermatocytes display long cilia (arrows). In contrast, Sas-4^F112A^ spermatocytes form cilia with reduced lengths. Asl (red) labels elongating centrioles and acetylated α-tubulin (green and arrows) labels primary cilia. Bar diagrams at right show the ciliary length measurements (*n*=40). ANOVA, ***P<0.0001. Error bars represent±s.e.m. Scale bar, 1 μm. Number of experiments (N)=3. Scale bar, 1 μm (**d**) Hypothetical model depicts how Sas-4^EE78RR^ differs from Sas-4^WT^ in promoting centriolar microtubule growth. Various domains of Sas-4 are shown. Red dots mark the mutations studied at the N- and C-terminus of PN2-3. Microtubule-binding domain (MBD) and TCP domain that are C-terminus to PN2-3 are shown.

We then performed EM analyzes of Sas-4^F112A^ centrioles to analyze centriolar architecture when Sas-4-tubulin interaction is significantly perturbed. In contrast to Sas-4^WT^ and Sas-4^EE78RR^, the spermatogonium and spermatocyte centrioles of Sas-4^F112A^ mostly contained doublet instead of triplet centriolar-microtubules (Fig. 6a,b). This finding indicated that Sas-4^F112^-tubulin interaction is not required for nucleating centriolar-microtubules *per se* but for the formation of third centriolar-microtubules suggesting insufficient tubulin delivery by perturbed Sas-4-tubulin interaction. Indeed, γ-TuRC has been suggested to nucleate the A-microtubule that subsequently templates B-and C-microtubule elongation^31^.

When analyzing primary cilia of spermatocytes, we identified that flies expressing Sas-4^EE78RR^ resulted in long cilia. In contrast, spermatocyte cells expressing Sas-4^F112A^ displayed shorter cilia (Fig. 6c). Furthermore, EM analyses revealed that Sas-4^F112A^ cilia displayed incomplete and distorted ciliary microtubules (Fig. 6e). These findings indicate that Sas-4-tubulin interaction is required to form structurally stable and defined lengths of ciliary microtubules. Taken together, these studies indicate that via interacting with tubulin, CPAP/Sas-4 proteins have a conserved function to define centriolar- and ciliary-microtubule lengths.

## Discussion

In cells, how a fraction of specialized tubulin from a large cytoplasmic pool is specifically recognized and licensed to form defined numbers and lengths of centriolar- and ciliary-microtubules is poorly understood. In this work, we have undertaken an integrated approach to dissect the conserved function of CPAP-tubulin interaction, which represents a crucial step in understanding centriolar- and ciliary-microtubule length control. Our results support a model in which CPAP/Sas-4 through the different facets of its PN2-3 domain, defines centriolar- and ciliary-microtubule lengths. Our structural studies reveal that PN2-3 embraces a ‘necklace-like' mode for tubulin recognition, in which, CPAP via its PN2-3_N_ targets the lumen surface of β-tubulin, and PN2-3_C_ loop-helix motif occupies the β-tubulin microtubule outer surface (Fig. 2d). This unique binding mode practically approves the formation of a high-affinity CPAP-GTP-tubulin complex and simultaneously prevents the polymerization of its bound GTP-tubulin, which is reactive, unstable, and ready for microtubule build-up once released. Noticeably, the last eight C-terminal residues of PN2-3_C_ were not visible in the electron density map. However, these residues together with F385, seem to play important roles in microtubule depolymerization, as the PN2-3_C2_ variant with truncation of its last ten residues, almost failed to depolymerize GTP-stabilized microtubules, when compared with full length PN2-3_C_ (Supplementary Fig. 6a).

To our knowledge, CPAP is the first centrosomal protein shown to form a high-affinity complex with tubulin, making it unavailable for polymerization. Although, we do not yet fully understand the mechanisms by which how the CPAP-bound tubulin is released at the sites of centriole assembly, it appears that controlled delivery of tubulin is essential for constructing defined numbers and lengths of centriolar- and ciliary-microtubules (Figs 3, 5 and 6). One of the potential mechanisms that could facilitate tubulin release at the site of centriole assembly is perhaps uncapping of CPAP's PN2-3_N_ from the β-tubulin surface. Thus, the cleared tubulin surface is then available for polymerization into micrometer scale centriolar-microtubules. This is supported by our experiments with CPAP^EE343RR^ / Sas-4^EE78RR^ and CPAP PN2-3_EX_^EE343RR^ variants with reduced tubulin affinity and unmasking of the β-tubulin surface causing overly long centriolar-microtubules (Figs 3d, 4c, 5b and 6d).

The finding that CPAP^EE343RR^ causes overly long daughter centrioles and enhances ciliary lengths without affecting mother centrioles suggests that CPAP specifically acts on growing microtubule-based structures. Although, these experiments indirectly suggest that CPAP could function as a centriolar-microtubule builder, currently we are unable to dissect centriole-specific CPAP functions. Thus, it is unknown how CPAP spatiotemporally exerts both its tubulin binding and polymerization activities. Since CPAP is a cell cycle regulated protein, it is likely that cell cycle-dependent posttranslational modifications and its interaction partners including centriolar-microtubule length determining factors are required to modulate CPAP's tubulin binding and polymerization activities^9^,^10^,^11^,^14^,^32^,^33^ As centriolar-microtubules markedly differ from cytoplasmic microtubules, experiments employing tools that allow inducible expression of centriole-specific CPAP or its microtubule bound atomic structure are additionally required to solve the puzzle of how CPAP acts on centriolar-microtubules.

CPAP^EE343RR^ expression causes enhanced ciliogenesis. This finding is strikingly similar to phenotypes observed when Kif24, a centriolar kinesin is depleted^33^. Interestingly, Kif24 specifically acts on mother centrioles and possesses microtubule-depolymerizing activity. Thus, it is tempting to speculate that an enhanced expression of CPAP^EE343RR^ could counteract endogenous Kif24 to promote ciliogenesis. Taken together, several questions remain, requiring future studies.

Although, our *in vitro* experiments suggest a possible role for CPAP functioning as an atypical centriolar-microtubule builder, our *in vivo* experiments in flies seem to support Sas-4's role mainly as a regulator of tubulin release at the onset of centriolar-microtubule elongation. The experiment that highlights this aspect is the proximal localization of endogenous Sas-4 at the elongating spermatocyte centrioles (Fig. 5b)^34^,^35^. This finding supports an idea that Sas-4 has a tendency to release tubulin to control centriolar-microtubule growth independent from its localization at the distal end of centrioles. CPAP at the distal end of the centriole could play a role in regulating the growing end of centriolar-microtubules (Figs 5c and 6d). The tubulin incorporation ability of Sas-4 is further supported by our EM experiments revealing doublet instead of triplet centriolar-microtubules in flies expressing Sas-4^F112A^, a variant that strongly reduces its interaction with tubulin. These findings not only support the tubulin incorporation aspect of Sas-4 but also highlight the requirement of other factors for centriolar-microtubule growth.

How does CPAP/Sas-4 play a role in ciliary microtubule growth control? It is known that during cilium construction, IFT machineries transport ciliary building blocks of tubulin from the cytoplasmic ciliary base to the tip^6^,^7^,^8^. As CPAP/Sas-4 mainly localizes to the ciliary base, it rules out a direct role for CPAP in ciliary microtubule elongation at the ciliary tip. Thus, CPAP/Sas-4 likely controls cilia growth indirectly by regulating the available pool of unpolymerized soluble tubulin at the ciliary base. This diffusible tubulin is then concentrated and transported by intraflagellar transport proteins for cilia elongation^6^,^7^,^8^. If this is true, there could be an interaction between CPAP-tubulin complex and a subset of IFT particles. Future experiments revealing these molecular interactions will help in linking cytoplasmic tubulin recognition and their subsequent transport within the cilium by IFTs.

As free tubulin is ubiquitously present in cells, this raises the possibility of forming increased numbers and lengths of centriolar and ciliary structures. However, cells have strict mechanisms to prevent this. One such mechanism is CPAP/Sas-4-tubulin in the cytoplasm. Under physiological conditions, CPAP/Sas-4 has lower expression levels in contrast to free tubulins in cells^15^. Thus, at equilibrium, the amount of cytoplasmic tubulin bound by CPAP/Sas-4 making it unavailable for polymerization would only be a small fraction of the free tubulin pool. Therefore, it makes sense that CPAP/Sas-4 has a dedicated function in binding limited amounts of cytoplasmic tubulin and licenses them for centriolar- and ciliary-microtubules, which is then spatiotemporally regulated for constructing defined numbers and lengths of centriolar and ciliary structures.

## Methods

### Protein and peptide preparation

The cDNA encoding human CPAP (UniProtKB: Q9HC77) PN2-3 domain (aa 319-394) was cloned into a modified pRSFDuet vector with an N-terminal 10xHis tag followed by a PreScission^Tm^ protease cleavage site. Recombinant wild type and mutant PN2-3 proteins were overexpressed in *E. coli* BL21 (DE3). After overnight induction by 0.2 mM isopropyl β-D-thiogalactoside (IPTG) at 20 °C in LB medium, cells were harvested and suspended in buffer: 20 mM Tris, 200 mM NaCl, pH 8.0. Then cells were lysed with an Emulsiflex C3 (Avestin) high-pressure homogenizer. After centrifugation at 32,000 × *g*, the supernatant was applied to a HisTrap column (GE Healthcare), and the protein was eluted with a linear imidazole gradient from 50 to 500 mM. The resultant protein was digested overnight by PreScission protease to remove 10xHis tag, and further purified by HiTrap Heparin HP and Superdex 200 columns (GE Healthcare). Both wild type and mutant PN2-3 proteins were concentrated to 1 mM in 1xBRB80 buffer (80 mM PIPES-K, 1 mM MgCl_2_, 1 mM EGTA, pH 6.8), and stored at −80 °C for future use.

Human CPAP PN2-3_EX_ (aa 121-1060) was cloned into the pMlink vector (gifted from Yigong Shi's lab) with an N-terminal 2xStrep-Flag tag, and point mutations were further introduced following the same method described above. Recombinant wild type and mutant PN2-3_EX_ proteins were expressed in HEK 293F cells (Invitrogen), which cultured in SMM 293 T-I medium (Sino Biological Inc.) with shaking (3.5 × *g*) at 37 °C under 5% CO_2_ and 75% humidity. When cell density reached 1.5 × 10^6^ cells per ml, wild type or mutant PN2-3_EX_ plasmids were transfected into cells using titrated concentration of 25-KDa linear polyethylenimines (PEIs) (Polysciences). After 60 h from transfection, cells were harvested by centrifuge at 800 × *g* and resuspended in a lysis buffer containing 20 mM Tris, 200 mM NaCl, pH 8.0, and protease inhibitor cocktails (Amresco). Cell lysis was conducted by sonication on ice, and the suspension was centrifuged at 38,000 × *g* for 1 h to remove any cell debris. The supernatant was incubated with Strep-Tactin Superflow (IBA BioTAGnology) beads for 30 min at 4 °C. The resin was washed three times, each with 10 ml lysis buffer, and wild type or mutant PN2-3_EX_ proteins were finally eluted with lysis buffer supplied with 10 mM d-Desthiobiotin (Sigma).

The codon-optimized DARPin cDNA was assembled from a dozen of DNA oligos following a reported method^36^, and then cloned into the pRSFDuet vector (Novagen) with an N-terminal 6xHis tag. Recombinant DARPin was overexpressed in *E. coli* BL21 (DE3) by overnight induction using 0.2 mM IPTG at 20 °C. After cell harvest and lysis, the 6xHis DARPin protein was purified to homogeneity over successive HisTrap, anion exchange Q, and Superdex 200 columns (GE Healthcare). The protein was concentrated to 1 mM in 1xBRB80 buffer and stored at −80 °C for future use.

Recombinant GFP-tagged fly EB1 protein was expressed in *E. coli* Bl21 (DE3) and prepared following the method reported previously^26^ with minimum modifications.

Peptides of PN2-3_N_ (aa 323-361), PN2-3_C_ (aa 372-394), and other PN2-3_C_ variants were synthesized in >95% purity by SciLight Biotechnology.

### Complex reconstitution and crystallization

Porcine/bovine brain tubulin was purified by modified procedures of two cycles of polymerization and depolymerization^37^. The purified tubulin was stored at −80 °C in 1xBRB80 buffer until use.

DARPin-tubulin complex was assembled by mixing DARPin and tubulin in a 1.5:1 molar ratio (DARPin to tubulin). The sample was incubated at 4 °C for 1 h and then loaded onto a pre-equilibrated Superdex 200 column (GE Healthcare) using 1xBRB80 buffer. Fractions responding to DARPin-tubulin complex were pooled and concentrated for future use.

DARPin-tubulin-PN2-3_C_ complex was reconstituted by mixing PN2-3_C_ with DARPin-tubulin complex in the molar ration of 1.2:1 (PN2-3_C_ to DARPin-tubulin) at a total concentration of 20 mg ml^−1^. After incubation at 4 °C for 1 h, crystallization screen was performed using an Art Robbins Gryphon crystallization robot by mixing equal volumes of DARPin-tubulin-PN2-3_C_ complex with different screening conditions from the commercial crystallization kits. Crystals appeared three days after tray set-up under 9 °C from the Hampton Research PEG/Ion crystallization screen kit. Manual crystallization and optimization were then conducted via the sitting-drop vapor diffusion method, and diffraction quality crystals were finally obtained under the reservoir condition of 0.2 M potassium sodium tartrate, 20% (w/v) polyethylene glycol 3,000, and 4% polypropylene glycol P 400.

### Data collection and structure determination

Crystals were briefly soaked in a cryo-protectant composed of reservoir solution supplemented with 20% glycerol, and were flash frozen in liquid nitrogen for data collection at 100 K. Data collection was performed at beamline BL17U at the Shanghai Synchrotron Radiation Facility under the wavelength of 0.9792 Å. Diffraction data were indexed, integrated and merged using the HKL2000 software package (http://www.hkl-xray.com/).

The structure of DARPin-tubulin-PN2-3_C_ was solved by the molecular replacement using MOLREP^38^,^39^ with the published DARPin-tubulin structure (PDB code: 4DRX) as search model. Structure refinement was performed using PHENIX^40^ with iterative manual model building using COOT^41^. In the final structure, PN2-3_C_ residues 372–386 were modelled. The last eight residues (387–394) are invisible due to flexibility. Data collection and structure refinement statistics were shown in Table 1. All structural figures were prepared using PYMOL (http://www.pymol.org/).

### Isothermal titration calorimetry

Calorimetric experiments were conducted at 15 °C with a MicroCal iTC200 instrument (GE Healthcare). All proteins and peptides, including tubulin, wild type and mutant PN2-3 proteins/peptides, were dialyzed against the 1 × BRB80 buffer (80 mM PIPES-K, 1 mM MgCl_2_, 1 mM EGTA, pH 6.8) prior to titration. Concentration of tubulin and PN2-3 proteins were determined by their respective absorbance at 280 nm. Peptides were quantified by weighing on a large scale and further confirmed and adjusted by their respective absorbance at UV_205_ *(14)*. Acquired calorimetric titration data were analyzed using Origin 7.0 (GE Healthcare) using the ‘One Set of Binding Sites' fitting model. In order to avoid microtubule formation, tubulin sample was kept under low temperature (<15 °C) and low concentration (<15 μM) during wild type and mutant PN2-3 titrations. Specifically, for ITC curves in Fig. 1d and Supplementary Fig. 3c, a sample cell tubulin concentration of 9 μM was used with resultant titration *C*-values ranging from 3.2 (PN2-3^F375A^) to 360 (PN2-3^WT^). For ITC titrations in Supplementary Fig. 1, a tubulin or DARPin-tubulin concentration of 36 μM was used to ensure reasonable titration *C*-value (0.5–10) and measurable heat signal.

### Microtubule pelleting assays

For microtubule co-sedimentation pelleting assays in Supplementary Figs 6 and 7, tubulin polymerization was first conducted at 37 °C using either GMPCPP or 33% (v/v) glycerol-containing buffer (80 mM PIPES-K, 5 mM MgCl_2_, 0.5 mM EGTA, 33% (v/v) glycerol). After centrifugation at room temperature, microtubules were harvested and re-suspended in warm 1 × BRB80 buffer. Different amount of PN2-3 proteins/peptides were then added and incubated at 37 °C for 20 min. After centrifugation under 100,000 × *g* at room temperature, all supernatant and pellet fractions were analyzed using SDS-PAGE.

### Cross-linking mass spectrometry

Equal molar of prepared PN2-3 and tubulin proteins were mixed and incubated at 25 °C for 10 min. 5- to 80-fold excess BS^3^ was used to perform cross-linking reaction between PN2-3 and tubulin. After incubate reaction systems at 25 °C for 1 h, final concentration of 1 mM Tris base was added to stop reaction. The resultant sample was then applied to SDS-PAGE, and the cross-linked band was excised for trypsin digestion with or without prior reduction and alkylation in 50 mM ammonium bicarbonate at 37 °C overnight.

The peptides were extracted twice with 1% trifluoroacetic acid in 50% acetonitrile aqueous solution for 30 min. The extractions were then centrifuged in a speedvac to reduce the volume. For LC-MS/MS analysis, the digestion product was separated by a 65 min gradient elution at a flow rate 0.25 μl min^−1^ with the EASY-nLCII integrated nano-HPLC system (Proxeon, Denmark), which was directly interfaced with the Thermo Q Exactive mass spectrometer. The analytical column was a home-made fused silica capillary column (75 μm ID, 150 mm length; Upchurch, Oak Harbor, WA) packed with C-18 resin (300 A, 5 μm, Varian, Lexington, MA). Mobile phase A consisted of 0.1% formic acid, and mobile phase B consisted of 100% acetonitrile and 0.1% formic acid. The Q Exactive mass spectrometer was operated in the data-dependent acquisition mode using the Xcalibur 2.0.7 software and there is a single full-scan mass spectrum in the Orbitrap (400–1800 m z^−1^, 30,000 resolution) followed by 20 data-dependent MS/MS scans at 35% normalized collision energy. The MS/MS spectra for cross-linked peptides from each LC-MS/MS run were searched against the selected database using the pLink program^42^.

### Data availability

Coordinates have been deposited at the Protein Data Bank under accession code 5EIB. The data that support the findings of this study are available from the corresponding author upon request.

## Additional information

**How to cite this article:** Zheng, X. *et al.* Molecular basis for CPAP-tubulin interaction in controlling centriolar and ciliary length. *Nat. Commun.* 7:11874 doi: 10.1038/ncomms11874 (2016).

## Supplementary Material

Supplementary Information Supplementary Figures 1-12, Supplementary Table 1, Supplementary Methods and Supplementary References

Supplementary Movie 1 Microtubule depolymerization assay of wild type PN2-3 showing its capability in microtubule sequestration. The movie was recorded on TIRF (total internal reflection fluorescence) microscopy (NikonEclipse Ti) within 10 min after adding PN2-3^WT^.

Supplementary Movie 2 Microtubule depolymerization assay of F375A PN2-3 mutant which is unable to depolymerize microtubule. The movie was recorded on TIRF microscopy (NikonEclipse Ti) within 10 min after adding PN2-3^F375A^.

Supplementary Movie 3 Microtubule depolymerization assay of EE343RR PN2-3 mutant showing their dynamic capability in microtubule sequestration. The movie was recorded on TIRF microscopy (NikonEclipse Ti) within 10 min after adding PN2-3^EE343RR^.

Supplementary Movie 4 Live cell imaging of wild type CPAP (CPAP^WT^)expressing RPE1 cells. Centrioles of control cells stably expressing CPAP-GFP were recorded by time-lapse fluorescence microscopy using 63x oil objective. Square highlights CPAP^WT^ centrioles. Centrioles of cells expressing CPAP^WT^ remain constant over a period of time, which is known to be approximately 500 nm. Frames were taken every 5 min. Time counter shows hr:min.

Supplementary Movie 5 Second example of live cell imaging. Centrioles of control cells stably expressing CPAP-GFP were recorded by time-lapse fluorescence microscopy using 40x water objective. Frames were taken every 5 min. Time counter shows hr:min.

Supplementary Movie 6 Live cell imaging of CPAP^EE343RR^ expressing RPE1 cells showing a rapid rate of centriolar elongation. Centrioles of cells stably expressing CPAP^EE343RR^-GFP were recorded by time-lapse fluorescence microscopy using 40x water objective. Movie starts with an interphase cell. At the end of the movie, centriole separation is visible. Square highlights CPAP^EE343RR^ centrioles. Frames were taken every 5 min. Time counter shows hr:min.

Supplementary Movie 7 Second example of live cell imaging. Centrioles of cells stably expressing CPAP^EE343RR^-GFP were recorded by time-lapse fluorescence microscopy using 40x water objective. Movie starts with a mitotic cell in which the centrioles from both of the daughter cells are imaged. At the end of the movie, centriole separation is visible. Square highlights CPAP^EE343RR^ centrioles. Note that the centrioles of cells expressing CPAP^EE343RR^ rapidly elongate within a short period of time and remains over a longer period of time reaching up to 3.5μm. Frames were taken every 5 min. Time counter shows hr:min.

Supplementary Movie 8 TIRF-based microtubule polymerization experiment using buffer control. The movie was recorded on TIRF microscopy (NikonEclipse Ti) within 5 min after adding control buffer.

Supplementary Movie 9 TIRF-based microtubule polymerization experiment of wild type PN2-3_EX_. The movie was recorded on TIRF microscopy (NikonEclipse Ti) within 5 min after adding PN2-3_EX_^WT^.

Supplementary Movie 10 TIRF-based microtubule polymerization experiment of F375A PN2-3_EX_ mutant. The movie was recorded on TIRF microscopy (NikonEclipse Ti) within 5 min after adding PN2-3_EX_^F375A^.

Supplementary Movie 11 TIRF-based microtubule polymerization experiment of EE343RR PN2-3_EX_ mutant. The movie was recorded on TIRF microscopy (NikonEclipse Ti) within 5 min after adding PN2-3_EX_^EE343RR^.

## Figures and Tables

**Table 1:** Data collection and refinement statistics.

	DARPin-Tubulin-PN2-3_C_
*Data collection*	
Space group	P2_1_
Cell dimensions	
*a*, *b*, *c* (Å)	73.9, 91.1, 83.3
α, β, γ (°)	90, 97, 90
Wavelength (Å)	0.9792
Resolution (Å)	50–2.1 (2.17–2.10)*
*R*_merge_ (%)	12.8 (89.9)
*I*/σ*I*	16.8 (2.5)
Completeness (%)	99.1 (98.2)
Redundancy	4.6 (4.6)
	
*Refinement (F>0)*	
Resolution (Å)	50–2.1
No. reflections	63,551
*R*_work_/*R*_free_ (%)	17.8/22.2
No. atoms	
Protein	8021
Ligand	66
Water	597
*B*-factors (Å^2^)	
Protein	29.6
Ligand	21.5
Water	35.3
r.m.s. deviations	
Bond lengths (Å)	0.01
Bond angles (°)	1.26

^*^Values in parentheses are for highest-resolution shell.
