# Supplementary material for: Molecular basis for CPAP-tubulin interaction in controlling centriolar and ciliary length
Source: Nat Commun. 2016 Jun 16;7:11874. doi: 10.1038/ncomms11874 (PMC4912634; doi:10.1038/ncomms11874)
Supplement: Supplementary Information — Supplementary Figures 1-12, Supplementary Table 1, Supplementary Methods and Supplementary References [file ncomms11874-s1.pdf]

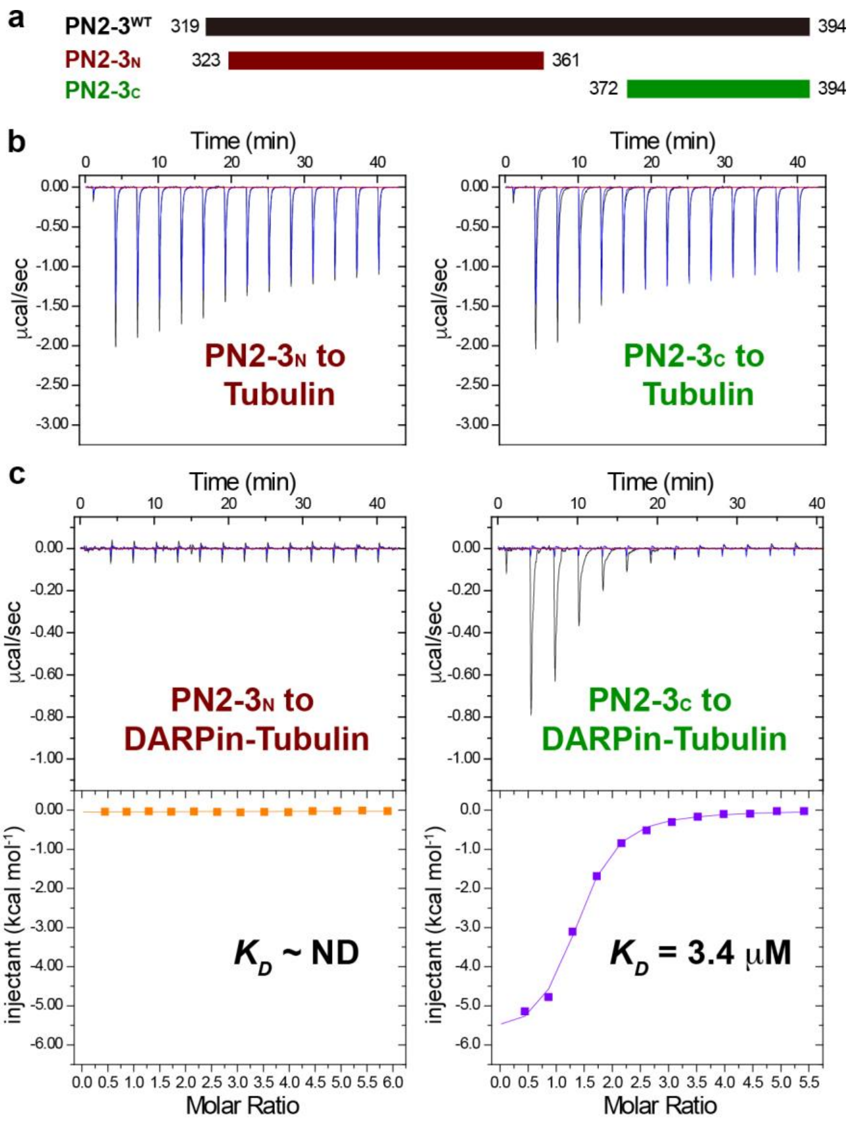

Supplementary Figure 1. DARPin and PN2-3$_C$ are compatible for tubulin dimer binding. (a) Diagrams of wild type PN2-3 (PN2-3$_{WT}$), PN2-3$_N$ and PN2-3$_C$. **(b)** ITC titration curves of PN2-3$_N$ (1 mM) and PN2-3$_C$ (1mM) titrated into tubulin dimer (36 μM). **(c)** ITC titration and fitting curves PN2-3$_N$ (1 mM) and PN2-3$_C$ (1mM) titrated into DARPin-tubulin dimer (36 μM). Background titrations of buffer to tubulin in **(b)** and **(c)** were shown as blue curves and overlaid to their respective peptide sample titrations. Peptide to buffer titrations yielded no heat signal (data not shown). The background heat signals were subtracted for ITC curve fitting. The presence of DARPin completely inhibited PN2-3$_N$-tubulin but did not affect PN2-3$_C$-tubulin interactions, thus leading to the strategy of co-crystallization of the PN2-3$_C$-DARPin-tubulin complex.

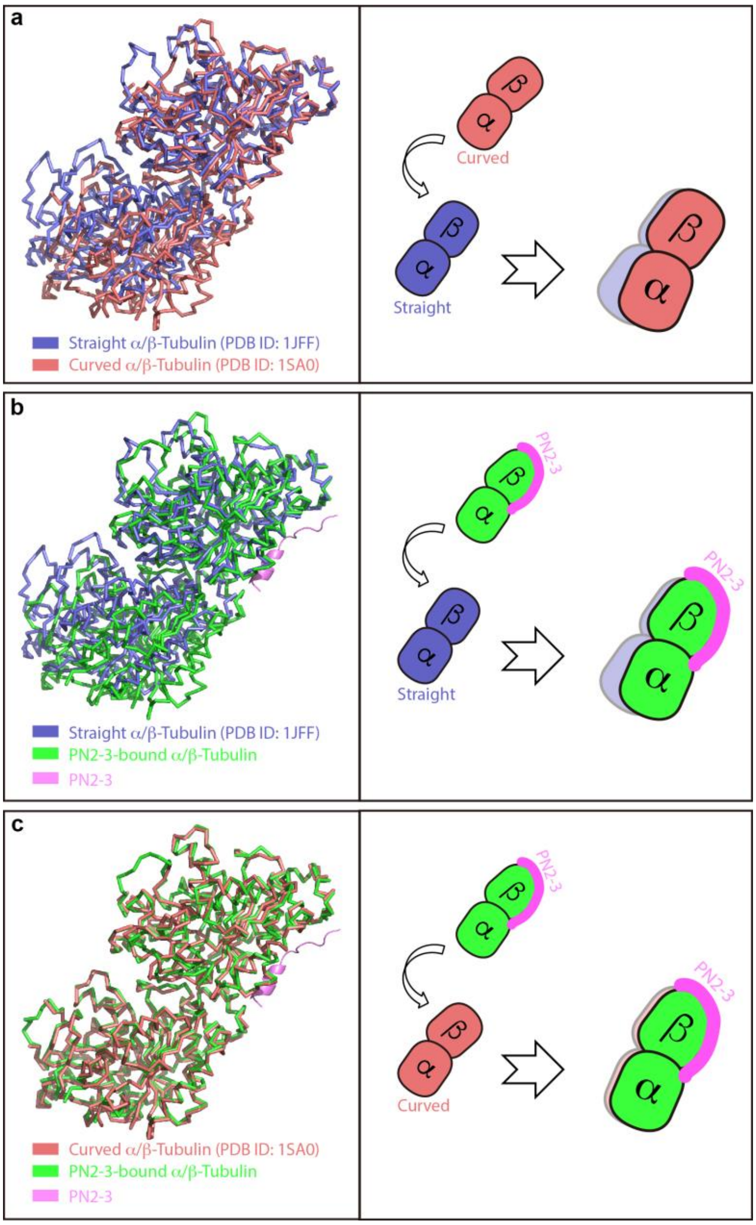

Supplementary Figure 2. Curved tubulin dimer is captured by PN2-3. (a) Left, structural alignment of a straight tubulin dimer (blue) as part of microtubule (PDB 1JFF) and a curved tubulin dimer (deep salmon) stabilized by a stathmin-like domain (PDB 1SA0). Structural superimposition is based on β-tubulin. Right, a cartoon diagram highlighting the imperfect superimposition between curved and straight tubulin dimers. **(b)** Structural alignment and cartoon diagram of the PN2-3$_C$–bound tubulin (green) superimposed with straight tubulin dimer (blue). Structural superimposition is based on β-tubulin. **(c)** Structural alignment and cartoon diagram of the PN2-3$_C$–bound tubulin (green) superimposed with curved tubulin dimer (deep salmon). The perfect superimposition suggests that PN2-3$_C$ binding to tubulin dimer favors a curved conformation.

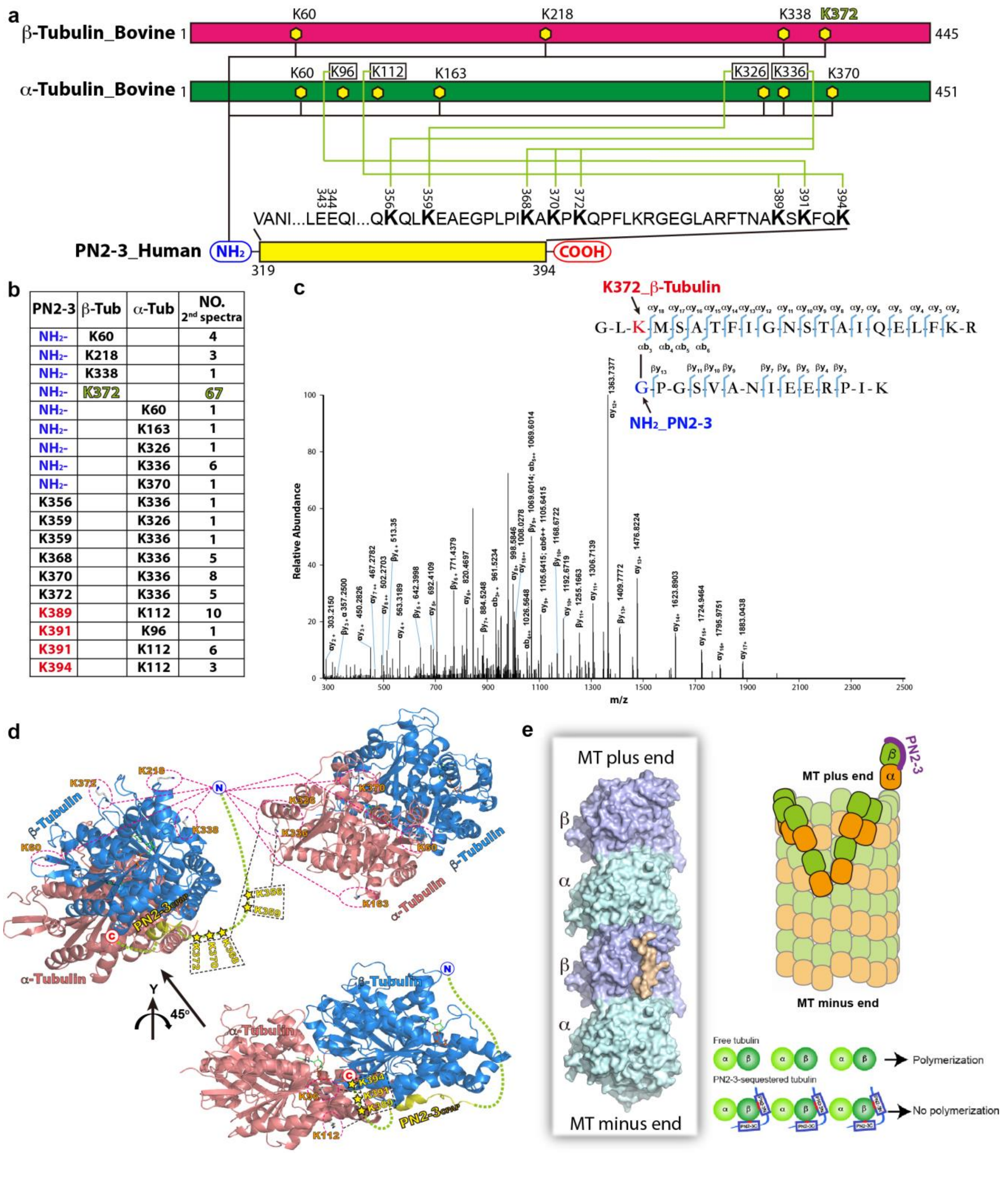

Supplementary Figure 3. Cross-linking mass spectrometry analysis of the PN2-3-tubulin complex. (a) Cross-link map for PN2-3 in complex with tubulin. The observed links from PN2-3 to tubulin dimer are denoted by direct lines. The identified cross-linking events within PN2-3$_N$ are mainly mediated by its N-terminal amine (black lines). A large number of links were identified between lysine residues within the PN2-3 “linker-PN2-3$_C$” region and tubulin (green lines). **(b)** Summary of

cross-links identified by MS. Number of the secondary mass spectra is also listed to indicate a relative abundance of the cross-links. Lysine residues highlighted in red belong to PN2-3_C C-terminal segment that is invisible in the crystal structure. **(c)** Example secondary mass spectrum of cross-linked NH2_PN2-3 and K372_β-tubulin. **(d)** Mapping of the cross-links over the structure of tubulin dimers. α-tubulin, β-tubulin, and PN2-3 are colored coded salmon, blue, yellow, respectively. Cross-links mediated by the N-terminal amine of PN2-3 map to the microtubule-lumen surface of β-tubulin within a tubulin dimer as well as the α-tubulin of an adjacent tubulin dimer, likely due to dynamic association of tubulin dimer with a tendency of microtubule formation. Despite invisible in crystal structure, the C-terminal lysine cluster of PN2-3_C forms multiple cross-links with α-tubulin lysine residues near the α-β-tubulin interface within a tubulin dimer, which is consistent with our crystal structure study and in part validates the cross-linking MS strategy. **(e)** Surface view of PN2-3_C bound to a tubulin dimer. PN2-3_C, β-tubulin, α-tubulin are colored wheat, light blue and pale cyan, respectively. PN2-3_C buries a solvent accessible surface area of 1212.7 Å^2 upon complex formation. On the right, positioning of PN2-3_C in the context of microtubule. Note, PN2-3_C binds to the microtubule outer surface in an anti-parallel orientation along the longitudinal axis of microtubule. Cartoon highlights how PN2-3 wraps around β-tubulin from microtubules outer surface as well as capping the β-tubulin surface, thus blocking its bound cytoplasmic tubulin dimer from polymerization.

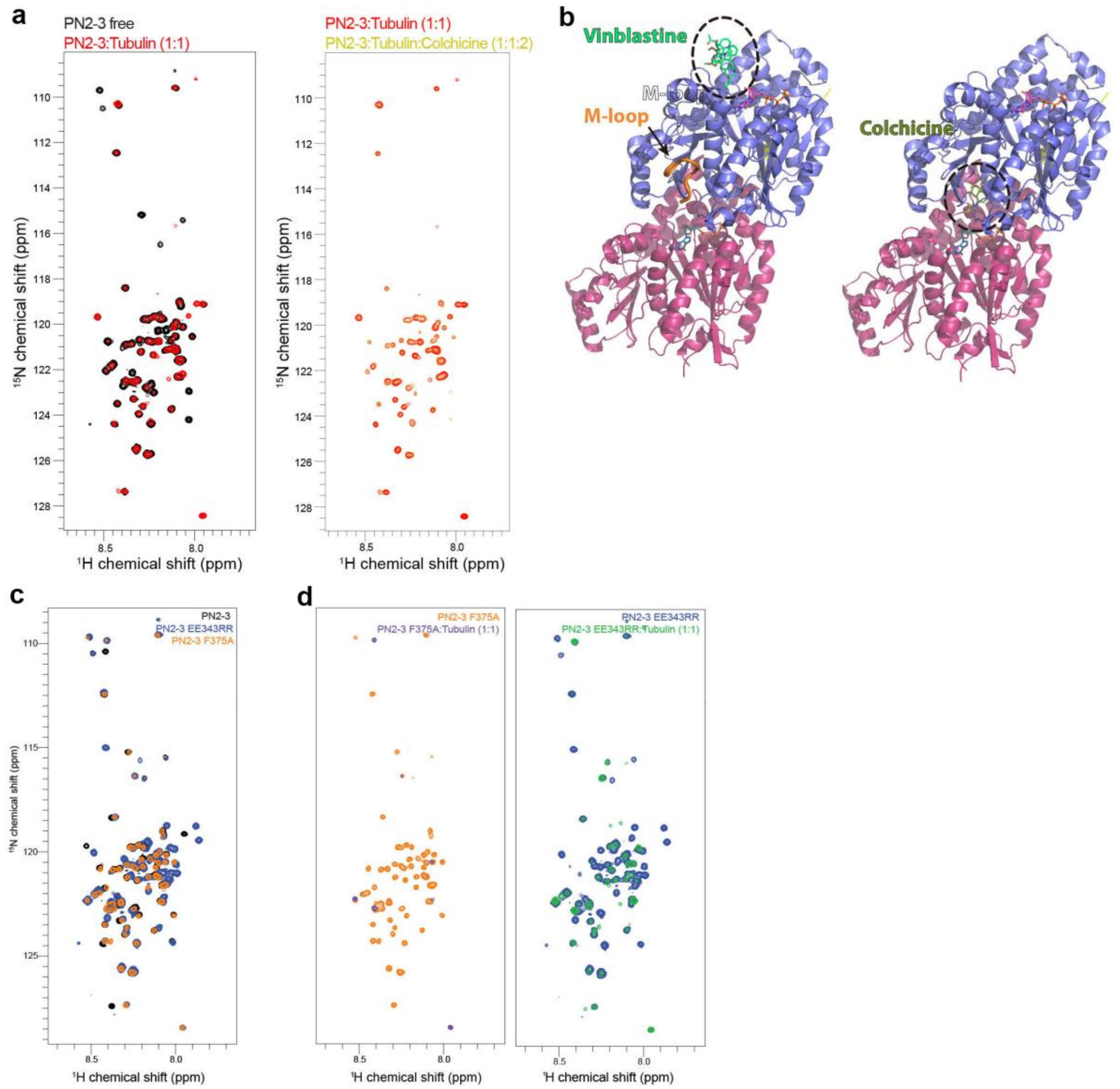

Supplementary Figure 4. Competitive NMR titration experiment to analyze PN2-3 and tubulin using vinblastine and colchicine. (a) Overlay of ^{1}H,^{15}N-SOFAST HMQC spectra of PN2-3-tubulin complex (red) alone, in the presence of 3-fold excess of Colchicine (golden), no detectable perturbation of PN2-3-tubulin interaction was observed as opposed to that of vinblastine **(Fig. 2f). (b)** Models display taxol, vinblastine and colchicine bound to tubulin dimers. **(c)** Overlay of ^{1}H,^{15}N SOFAST-HMQC spectra of PN2-3^{WT} (black), PN2-3^{F375A} (orange) and PN2-3^{EE343RR} (blue). Notably, the NMR spectra of PN2-3^{WT} and PN2-3^{F375A} are overall very similar while that of PN2-3^{EE343RR} is different. **(d)** Overlay of ^{1}H,^{15}N SOFAST-HMQC spectra of PN2-3^{F375A} and PN2-3^{EE343RR} in complex with tubulin at equimolar ratios are shown in purple (left panel) and green (right panel). The overall spectral quality of the complexes decreases in the order PN2-3^{WT} > PN2-3^{EE343RR} > PN2-3^{F375A} due to line broadening that is consistent with decreasing in affinity, where micromolar interactions may induce line-broadening of the NMR signals.

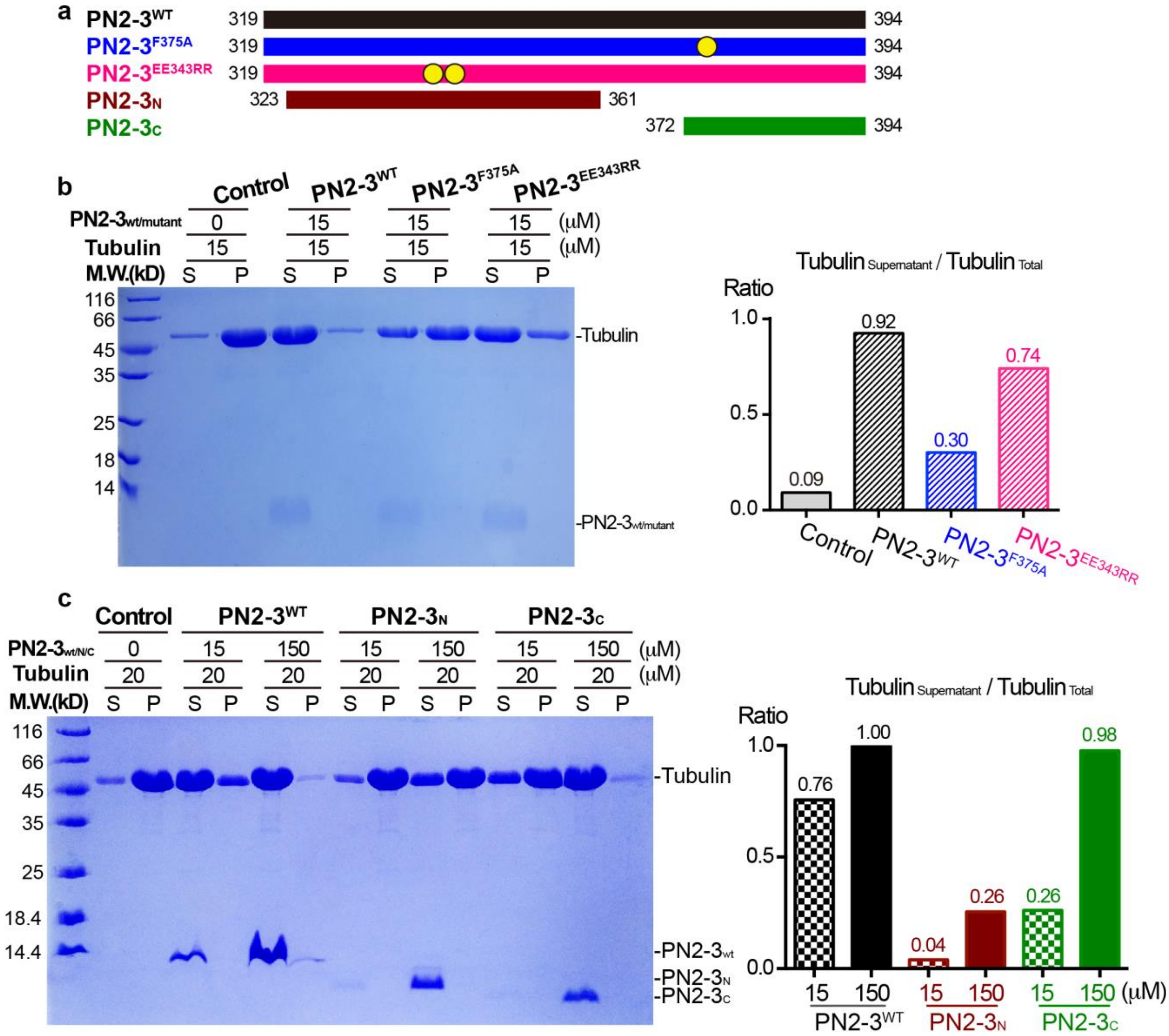

Supplementary Figure 5. CPAP PN2-3$_C$ via its F375 residue is sufficient to sequester tubulin from polymerization. **(a)** Diagram representing the PN2-3$_N$ and PN2-3$_C$ domain of CPAP. **(b)** PN2-3^{WT} and PN2-3^{EE343RR} prevents tubulin from polymerization. In contrast, PN2-3^{F375A} fails to do so indicating that the PN2-3's tubulin sequestering activity is F375 dependent. The ratio of tubulin in the supernatant to the total tubulin is represented in the right as a bar diagram. **(c)** Microtubule pelleting assay with PN2-3^{WT}, PN2-3$_N$ and PN2-3$_C$ domains. PN2-3$_C$ is sufficient to prevent tubulin from polymerization. The ratio of tubulin in the supernatant (S) to the total tubulin (S+P) is represented in the right as a bar diagram based on the densitometric quantification of the SDS-PAGE gel bands. The inhibitory efficiency of PN2-3$_{WT}$ at 150 μM is normalized to 1.

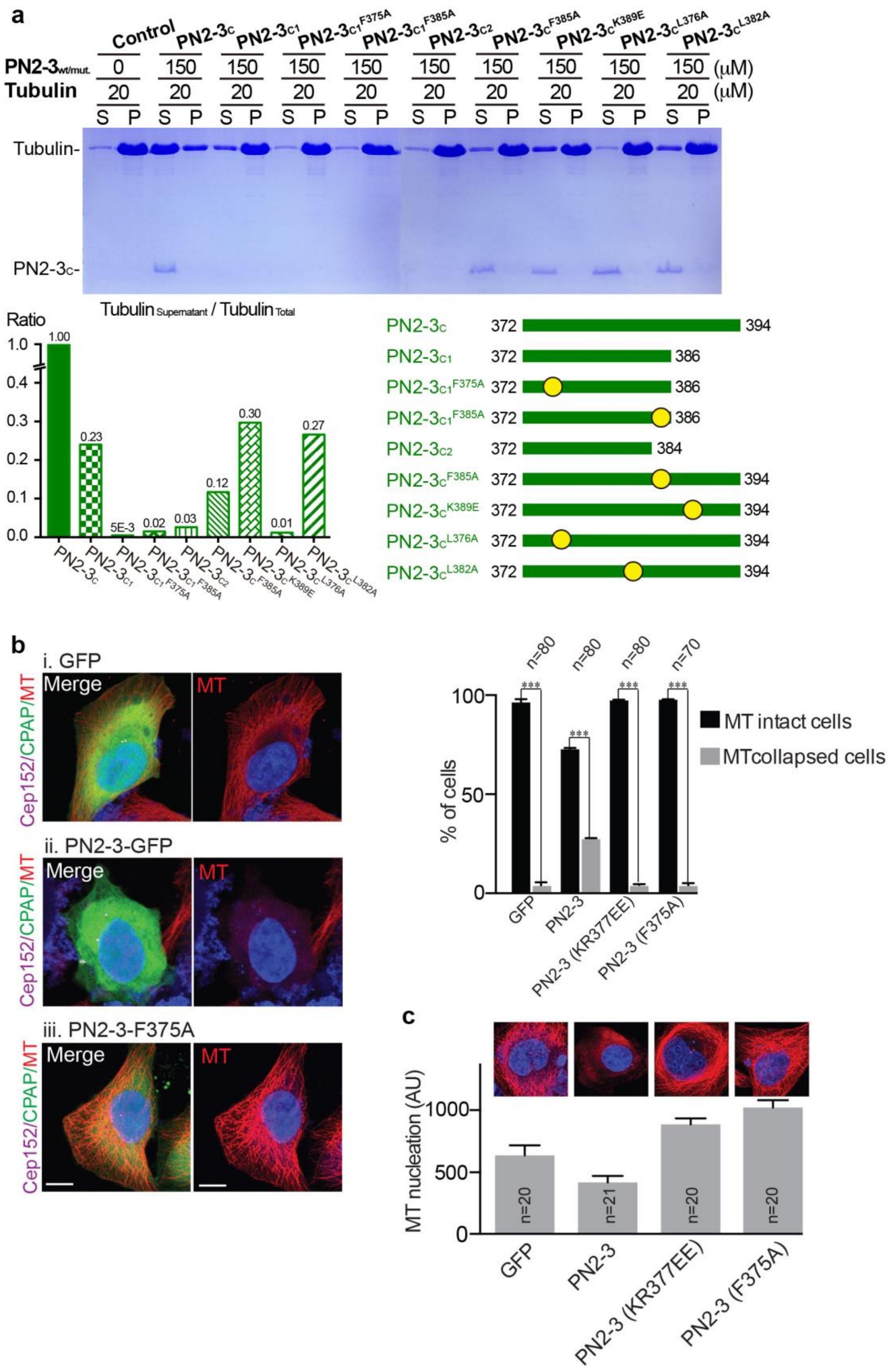

Supplementary Figure 6. PN2-3^{F375A} residue is essential for tubulin sequestration. (a) Microtubule pelleting assay with several truncated versions of the PN2-3$_C$ domain shows the significance of F375 residue in tubulin sequestration. The ratio of tubulin in the supernatant to the

total tubulin is represented in the right as a bar diagram. Note that while PN2-3_C exhibits the maximum tubulin sequestration activity (normalized to 1), PN2-3_{C1}^{F375A} displays the least activity. **(b)** Overexpression of GFP-tagged PN2-3^{WT} and PN2-3^{F375A} fragments in Hela cells. While PN2-3^{WT} fragment collapses the cytoplasmic microtubules, PN2-3^{F375A} prevents microtubules from collapsing eventually causing enhanced microtubule-nucleation in cells microtubules are labeled with anti-α-tubulin antibodies (red). Scale bar, 1 μm. Quantifications for percentages of cells with intact and collapsed microtubules (n>80 for all constructs except for PN2-3^{F375A}, where n=70, ANOVA, ***P<0.0001. Error bars represent ± s. e. m and the microtubules-nucleating intensities are given at right (n=20) **(c)**. Error bars represent ± s. e. m. Number of experiments (N)=3.

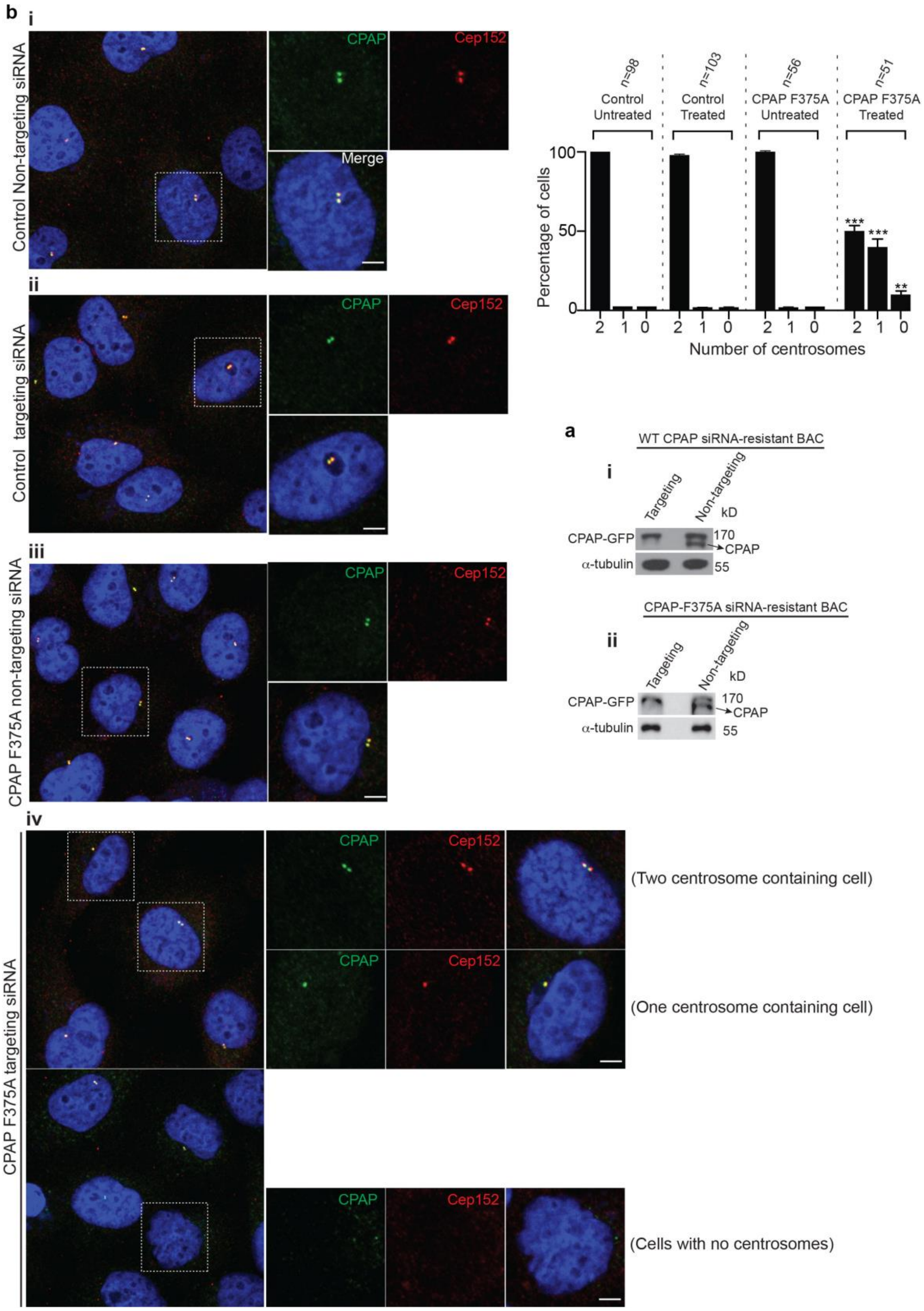

Supplementary Figure 7. CPAP-tubulin interaction is required for centrosome duplication in HeLa cells. (a) Western blots showing both wild type **(i)** and CPAP^{F375A} **(ii)** BACs are resistant for CPAP targeting siRNA, Note that endogenous CPAP is depleted in treatments with CPAP-targeting

siRNA. **(b)** Stable HeLa lines expressing RNAi-resistant GFP-tagged CPAP^{WT} **(i-ii)**, and CPAP^{F375A} **(iii-iv)** using artificial chromosome recombineering (BACs), a system that allows expressing gene products under their own endogenous promoters. As described previously, treating cells with CPAP-specific siRNA depleted endogenous protein, retaining the RNAi-resistant CPAP[1]. Expressing wild type siRNA-resistant CPAP^{WT} completely rescued the effect of siRNA-mediated CPAP depletion as cells proliferated without centrosome duplication defects **(i-ii)**. In contrast, RNAi-resistant CPAP^{F375A} failed to rescue the centrosome duplication phenotype caused by CPAP depletion **(iii-iv).** Specifically, when cultured for prolonged period of time, a proportion of cells displaying less than two centrosomes were noticed. Centrosomes are marked with CPAP (green) and Cep152 (red). Bar diagram quantifications showing percentages of cells showing centrosome numbers. (n>100 for CPAP^{WT}, n>50 for CPAP^{F375A}), ANOVA, , ***P<0.0001, , **P<0.001.Error bars represent ± s. e. m. Group of cells at low-magnifications are shown at left. Scale bar, 1 µm.

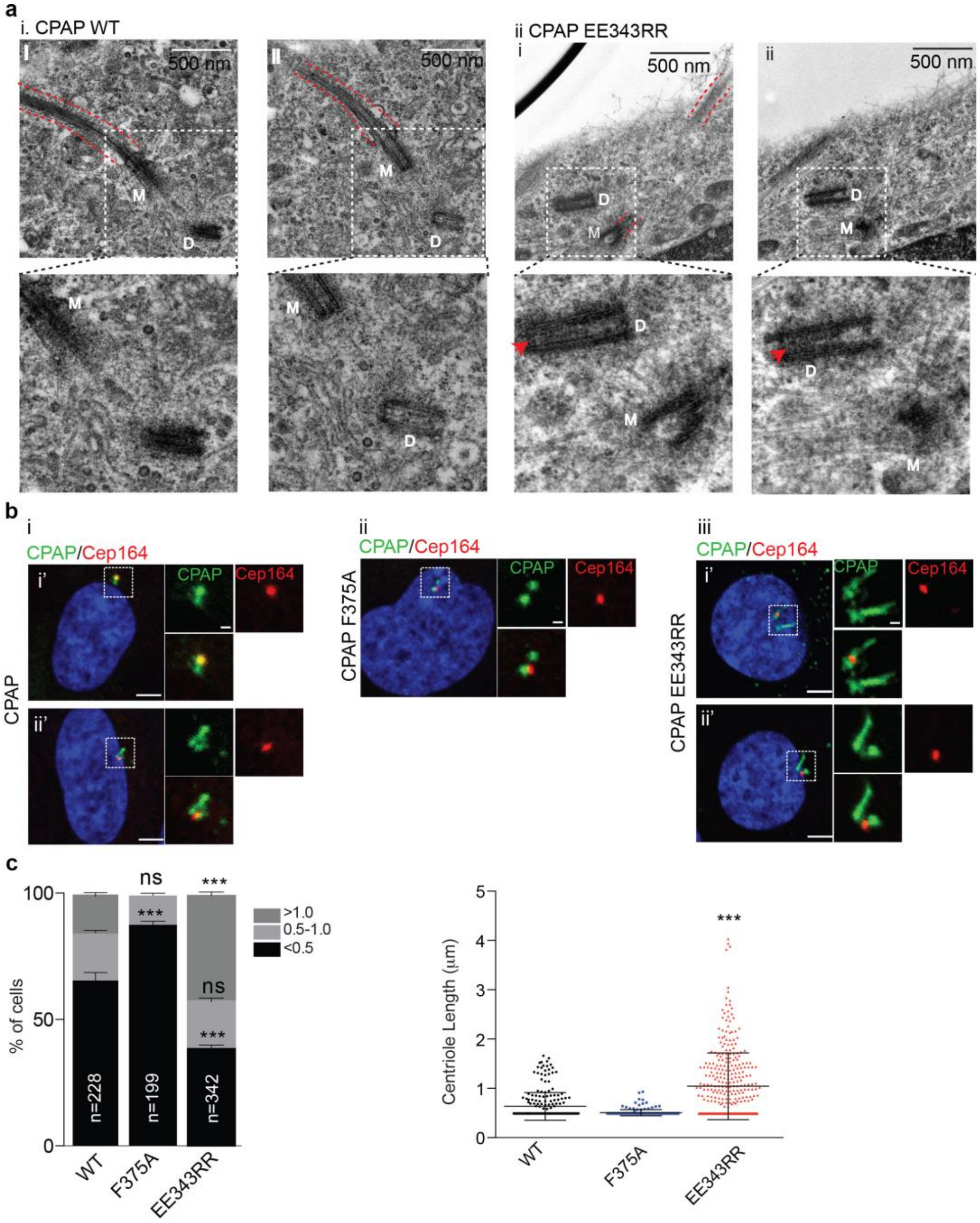

Supplementary Figure 8. CPAP^{EE343RR} expression causes overly long daughter centrioles. (a) Longitudinal serial sectioning EM-micrographs of RPE1 cells expressing CPAP^{WT} **(i)** and CPAP^{EE343RR} **(ii)**. In contrast to CPAP expression CPAP^{EE343RR} specifically causes lengthening of daughter centrioles (arrows). n>15. **(b)** RPE-1 cells expressing CPAP^{WT} **(i),** CPAP^{F375A} **(ii)** and CPAP ^{EE343RR} **(iii).** The CPAP variants are expressed as GFP (green), the mother centriole is labeled by centriolar appendage protein Cep164 (red) that specifies mother centrioles. Scale bar, 1 μm (insets 0.5 μm). **(c)** Quantification of mother centriole lengths. n=228 for WT, n=199 for CPAP^{F375A} and n=342 for CPAP^{EE343RR}. ANOVA, ***P<0.001, *P<0.05, Error bars represent ± s. e. m. Number of experiments (N)=3.

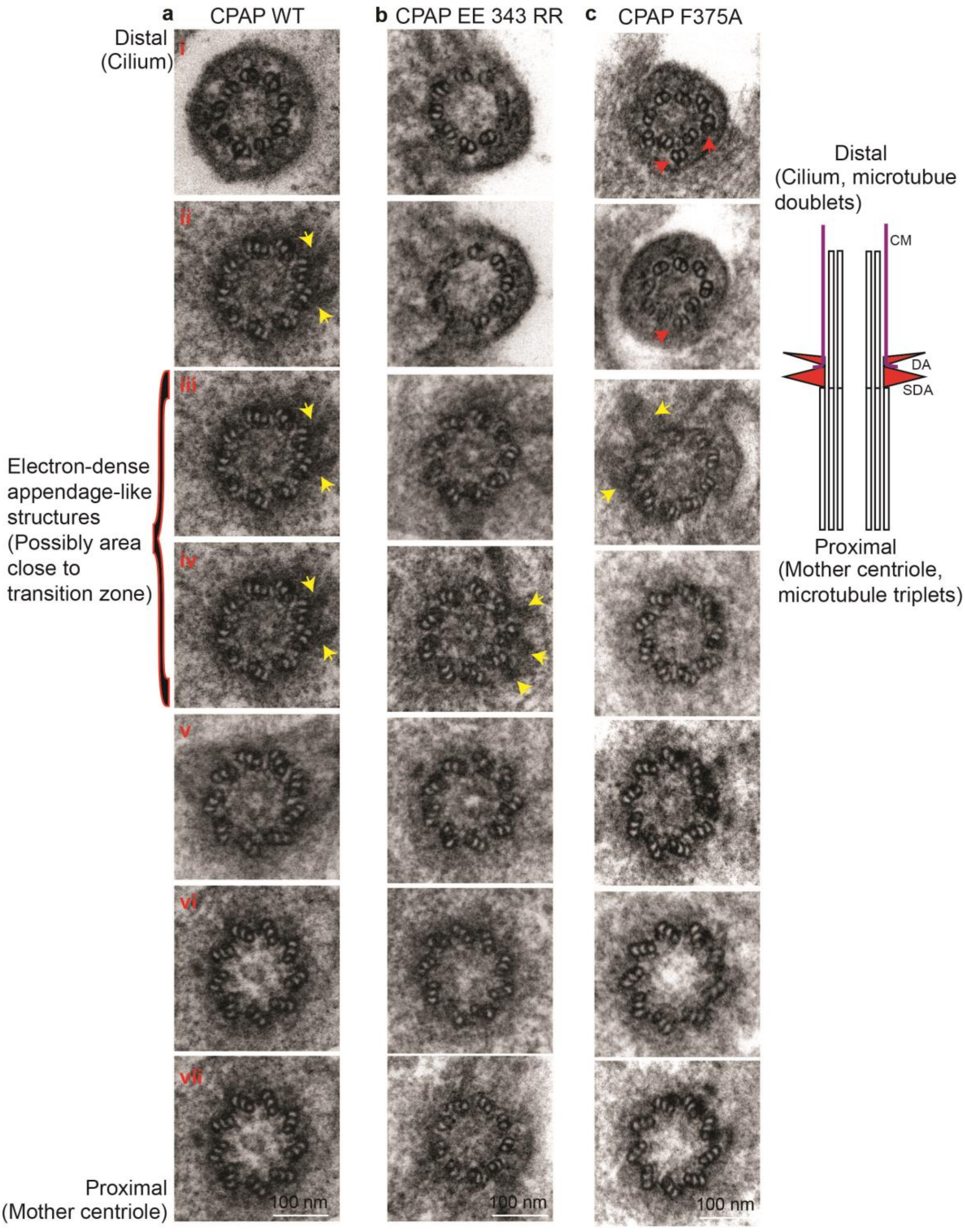

Supplementary Figure 9. CPAP^{EE343RR} expression causes overly long cilia. Cross serial sectioning EM-micrographs of RPE1 cells expressing CPAP^{WT} **(a),** CPAP^{EE343RR} **(b)** and CPAP^{F375A} **(c)**. Serial sections spans from proximal mother centriole (triplet microtubules) until the distal cilium (doublet microtubules). Electron dense area (yellow arrows) marks the region of ciliary transition zone. In contrast to CPAP^{WT} and CPAP^{EE343RR}, CPAP^{F375A} expression causes aberrant ciliary-microtubule organization. Scheme at right depicts centriolar and ciliary regions. (n=32)

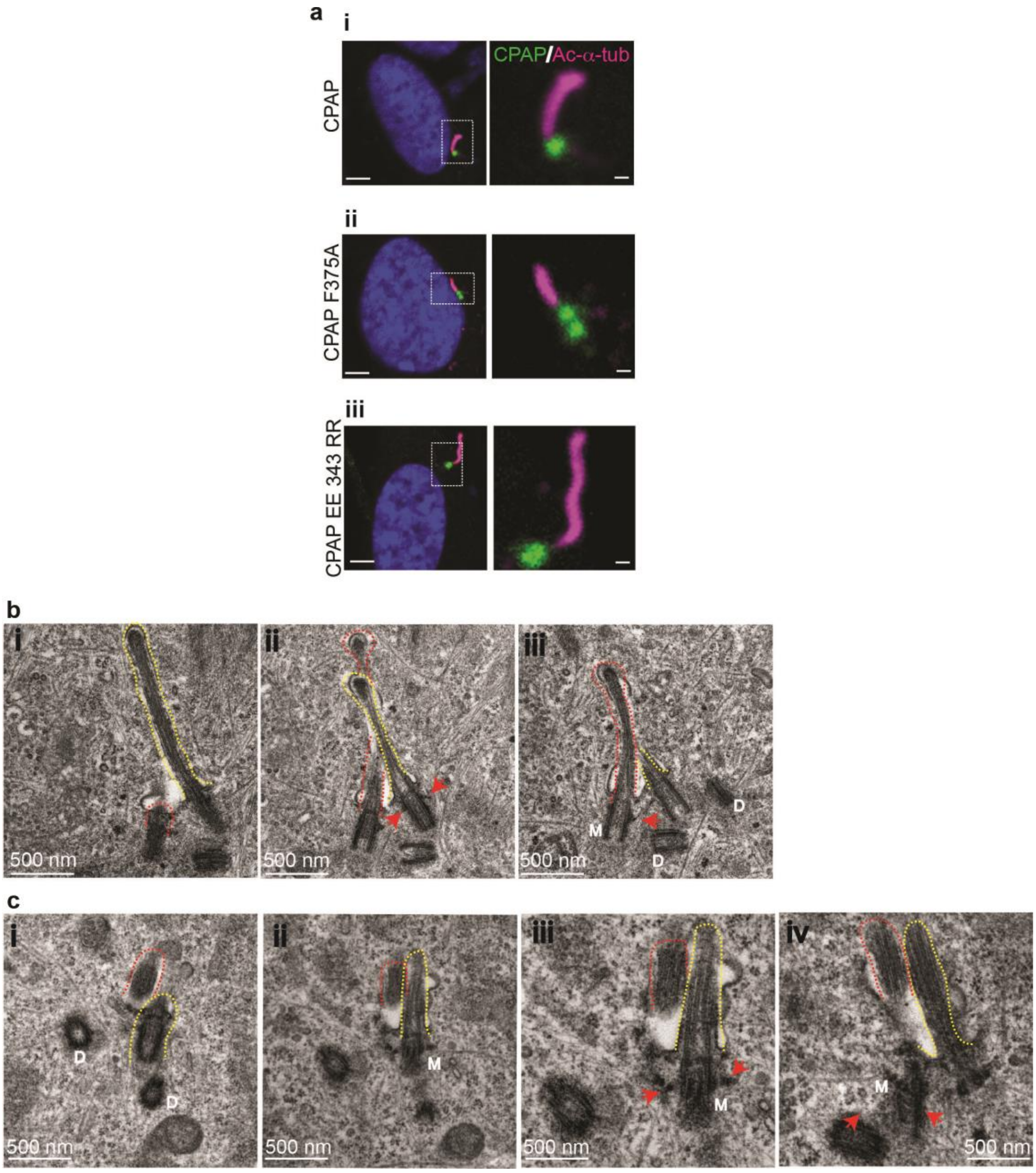

Supplementary Figure 10. CPAP^{EE343RR} expression causes overly long cilia and promotes daughter centrioles to form cilia. (a) RPE1 cells expressing CPAP^{WT} **(i),** CPAP^{F375A} **(ii)** and CPAP^{EE343RR} **(iii).** In contrast to CPAP^{WT} expression, CPAP^{EE343RR} causes cells to form long cilia. In contrast, CPAP^{F375A} expression causes cells to form short cilia. Cilia are labeled by acetylated α-tubulin (magenta). Scale bar, 1 μm (insets 1.0 μm). **(b-c)** Longitudinal serial sectioning-EM of at least two biciliated cells (dotted red and yellow lines). Four centrioles were observed and two of them constantly contained centriolar appendages (arrows) indicating that they are mother and matured daughter centrioles capable of forming cilia. (n=5).

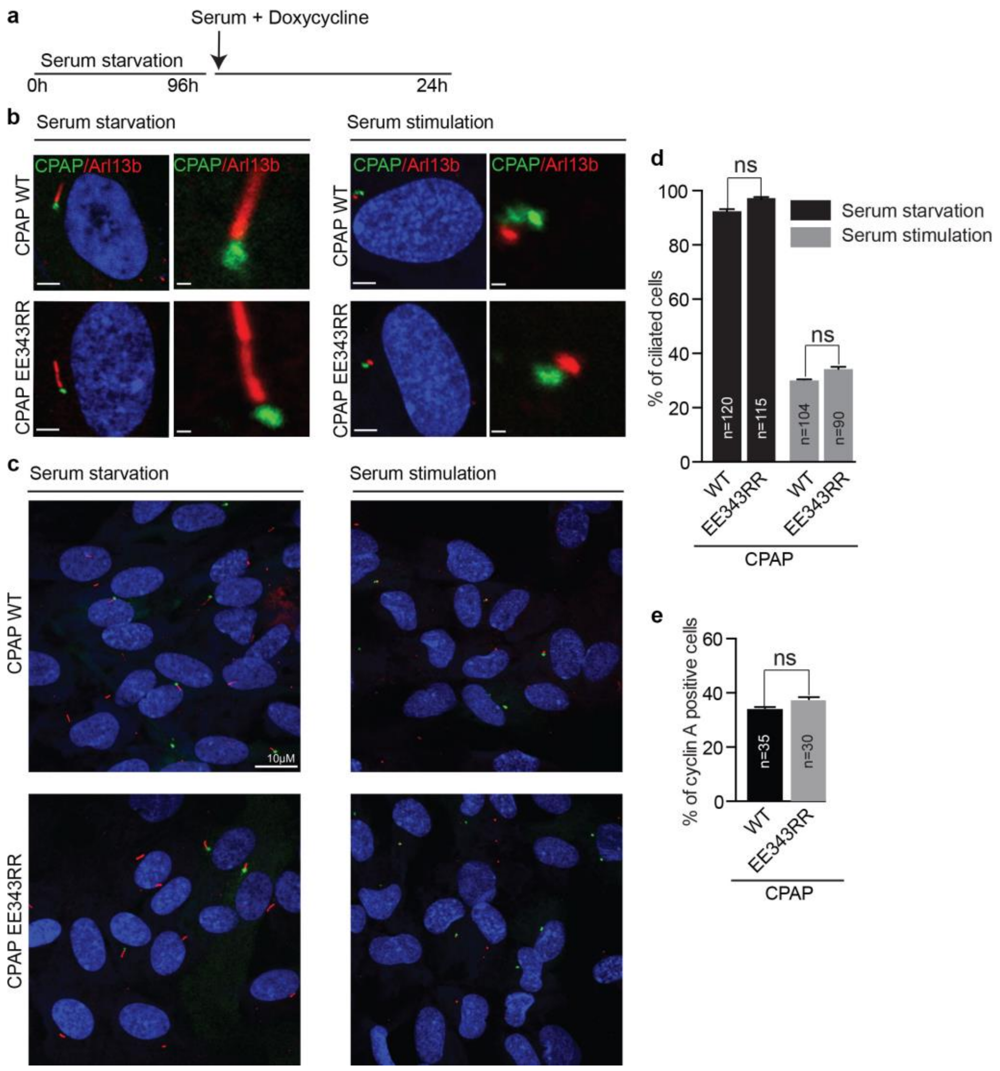

Supplementary Figure 11. Long cilia caused by CPAP^{EE343RR} expression is not due to defective cilia disassembly. (a) Experimental plan. RPE1 cells expressing doxycycline-inducible CPAP^{WT} or CPAP^{EE343RR} were synchronized at G_0-phase of cell cycle by serum starvation. A prolonged period of serum starvation for 96 hrs causes cells to have equivalent ciliary lengths. **(b)** Serum stimulation simultaneously with doxycycline-induction causes cells to disassemble cilia concurrent with cell cycle-re-entry. Scale bar, 1 µm (insets 1 µm). **(c)** Low-magnification overview of cells expressing CPAP^{WT} or CPAP^{EE343RR} cells after serum starvation and serum stimulation. Arl13b labels (red) cilia and ciliary remnants. Scale bar, 10 µm. **(d)** Quantification shows that CPAP^{EE343RR} cells disassemble cilia similar to CPAP^{WT} expressing cells and thus, there is no delay in cell cycle re-entry as measured by cyclin-A immunoreactivity **(e)**, a G1-S transition cell cycle marker. For cilia disassembly experiment: ANOVA, Error bars represent ± s. e. m. (n>100), number of experiments (N)=3. For Cyclin A experiment: (n>30), t-test, Error bars represent ± s. e. m. Scale bar, 1 µm.

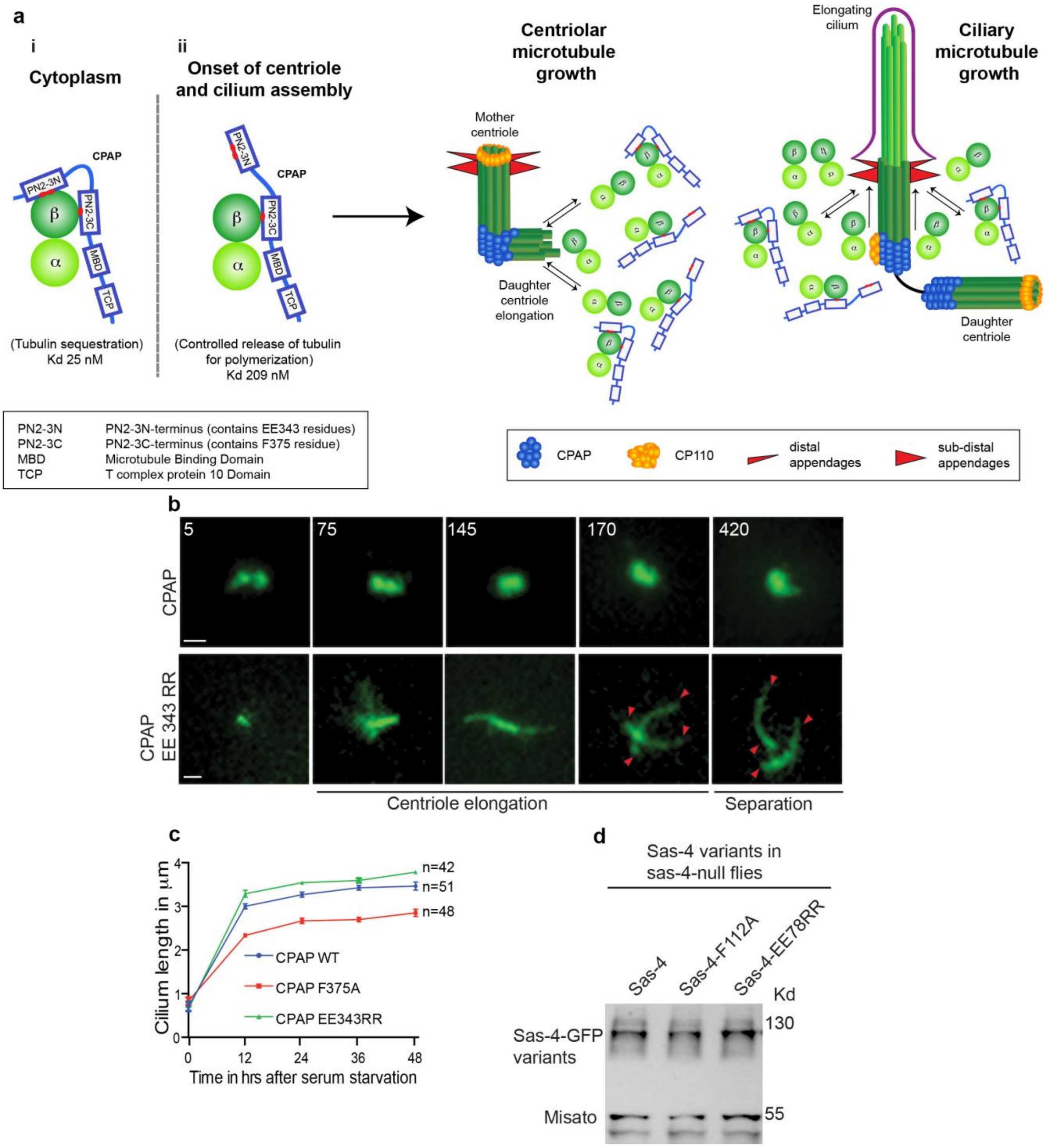

Supplementary Figure 12. A hypothetical model depicting how CPAP could control the delivery of its bound tubulin during centriolar and ciliary microtubule formation. (ai) In the cytoplasm, CPAP sequesters tubulin through its PN2-3 domain and forms a high affinity complex with a K_D of 25 nM. As given in **Fig. 1e**, while PN2-3_N caps the β–tubulins' lumen surface for polymerization, its PN2-3_C tethers β–tubulins from its outer surface. Different domains of CPAP are shown. **(aii)** At the onset of centriole and cilium assembly, CPAP delivers its sequestered tubulin by unmasking β–tubulin surface via its PN2-3N with a simultaneous reduction in binding affinity (K_D=209

nM). This could favor delivering of CPAP-bound tubulin available for centriolar and ciliary microtubule formation. CPAP's tubulin delivery could be regulated by cell cycle kinases, its interaction partners including centriolar- microtubule length determining factors [2-4]. Graphical depiction of centriolar appendages, CPAP and centriolar capping protein CP110 are painted in colors. **(b)** Time lapse imaging measurements of centriolar growth rate. A second example of RPE1 expressing $CPAP^{WT}$ and $CPAP^{EE343RR}$-GFP. While the CPAP expressing cell displays a controlled centriolar growth, $CPAP^{EE343RR}$, a mutation that perturbs PN2-3_N-mediated capping of β–tubulins' polymerization surface causes a rapid centriolar-elongation. In contrast to $CPAP^{WT}$-induced structures, elongating centriolar structures caused by $CPAP^{EE343RR}$ expression is distinct and measurable. Arrowheads mark fully elongated (at 170th mins) and separated (at 420th mins) centrioles. Scale bar is 1 μM. **(c)** Ciliary growth rate of RPE1 cells expressing $CPAP^{WT}$, $CPAP^{F375A}$ or $CPAP^{EE343RR}$. Ciliary lengths were measured at various time points after serum starvation. Note, experiments began with asynchronously growing cells. Compared to $CPAP^{WT}$, cells expressing $CPAP^{EE343RR}$ display an enhanced ciliary growth rate and cells expressing $CPAP^{F375A}$ display a reduced ciliary growth rate (n>40), Error bars represent ± s. e. m. **(d)** Western blots showing nearly same level of Sas-4 variants are expressed in Sas-4 null flies. Each lane contains equivalent numbers of testes. Misato is used as a loading control.

Supplementary Table 1

Summary of thermodynamic parameters from ITC

Tubulin	PN2-3$^{WT/mut.}$	ΔH (kcal mol^{-1})	ΔS (cal mol^{-1} deg^{-1})	*K*a (M^{-1})	N
Tubulin	PN2-3^{WT}	-15.7±0.2	-19.6	3.9±1.6 E7	1.08±0.01
	PN2-3^{F338A}	-24.9±0.1	-54.5	8.7±0.6 E6	0.97±0.00
	PN2-3^{Y341A}	-14.6±0.1	-19.2	7.4±0.5 E6	1.19±0.00
	PN2-3^{ED339RR}	-15.9±0.1	-23.7	7.0±0.7 E6	0.98±0.01
	PN2-3^{EE343RR}	-10.9±0.1	-7.1	5.2±0.5 E6	1.20±0.01
	PN2-3^{EE349RR}	-6.4±0.1	8.0	3.9±0.3 E6	1.23±0.01
	PN2-3^{K372E}	-14.7±0.7	-21.9	2.4±0.7 E6	1.01±0.03
	PN2-3^{F375A}	-6.9±0.3	1.64	3.6±0.5 E5	1.00±0.00
	PN2-3^{K377E}	-14.0±0.2	-21.7	7.6±0.5 E5	1.13±0.01
	PN2-3^{R378E}	-16.4±0.3	-29.1	1.2±0.1 E6	1.20±0.02
	PN2-3^{R384E}	-17.1±0.6	-33.1	5.3±0.6 E5	1.19±0.03
	PN2-3^{F385A}	-20.8±0.3	-40.2	1.0±0.2 E7	1.13±0.01
	PN2-3$_N$	-6.0±1.0	-1.6	1.5±0.2 E4	1.25±0.18
	PN2-3$_C$	-8.0±0.1	-3.3	2.1±0.2 E5	1.28±0.02
DARPin-Tubulin	PN2-3$_N$	N.D.			
	PN2-3$_C$	-6.6±0.2	2.2	3.0±0.4 E5	1.13±0.02

PN2-3^{WT}: VANIEERPIKAAIGERKQTFEDYLEEQIQLEEQELKQKQLKEAEGPLPIKAKPKQPFLKRGEGLARFTNAKSKFQK

PN2-3$_N$: IQLEEQELKQKQLKEAEGPLPIKAKP

PN2-3$_C$: KQPFLKRGEGLARFTNAKSKFQK

N.D. not detected

Supplementary Methods

Microtubule end-tracking assay

Microtubule depolymerization assay was performed at room temperature by monitoring the catastrophe of GMPCPP-stabilized microtubules seeds, which were immobilized on coverslips using biotin-streptavidin links as previously described [5]. For preparation the reaction chambers, microscope slides and biotin-coated coverslips were assembled using double-side tape. After blocking reaction chambers with 1% Pluronic F127 and 0.5 mg ml^{-1} κ-casein for 5 min, 50 µg ml^{-1} streptavidin was flowed in and incubate for 5 min. Microtubule seeds assembled from 50 µM tubulin mixed with 10% rhodamine-labeled tubulin and 10% biotin-labeled tubulin under 1 mM GMPCPP were then specifically attached to the functionalized surface by previously bound streptavidin. After wash the chamber with 1xBRB80 buffer, microtubule severing was initiated by flowing in 15 µM PN2-3^{WT}/PN2-3^{F375A}/PN2-$3^{EE343RR}$ protein, which was diluted in 1xBRB80 buffer in presence of oxygen scavenger system (50 mM glucose, 400 µg ml^{-1} glucose-oxidase, 200 µg ml^{-1} catalase, and 4 mM DTT). After immediately seal the reaction chamber with candle wax, images were collected every 3 sec within 10 min using a TIRF (total internal reflection fluorescence) microscope (NikonEclipse Ti). ImageJ software (http://rsbweb.nih.gov/ij/) was used for kymograph presentation and image analysis[5].

Microtubule polymerization assay was conducted following the same method described above. Briefly, after pre-assembled microtubule seeds were attached to the functionalized reaction chambers, tubulin polymerizing was initiated by flowing in 1 µM PN2-3_{EX}^{WT}/PN2-3_{EX}^{F375A}/PN2-$3_{EX}^{EE343RR}$, 20 nM GFP-tagged EB1, 15 µM tubulin and 1.5 µM rhodamine-labeled tubulin, which were diluted in oxygen scavenger system supplied tubulin polymerizing buffer (80 mM PIPES-K, 150 mM KCl, 1 mM EGTA, 5 mM GTP, 4 mM $MgCl_2$ and 5% glycerol, pH 6.8). After seal the reaction chamber with candle wax, images were collected every 3 sec within 7.5min. A control of PN2-3_{EX}-null buffer was used to detect the autonomous tubulin polymerization in this assay.

NMR spectroscopy

All NMR spectra, to compare the WT with the mutant PN2-3 proteins were recorded at 298K on 800MHz or 900MHz Bruker spectrometer with concentrations PN2-3 (35µM):Tubulin (35 µM) for wildtype and PN2-3 mutant proteins (F375A, EE343RR). The inhibitors (vinblastine

and colchicine) were added in 3-fold molar excess. The buffer used in all experiments is 50mM sodium phosphate, 100mM NaCl, 1mM EGTA, 1mM $MgCl_2$, 1mM GDP pH 6.8.

Plasmids and cloning for cell culture experiments

PN2-3^{WT}, PN2-$3^{KR377EE}$ and PN2-3^{F375A} encompassing the regions (aa 317-394) were cloned into pEGFP-C1 vector for transient transfection in HeLa cells. $CPAP^{WT}$, $CPAP^{F375A}$ and $CPAP^{EE343RR}$ genes were cloned into pSinEF-2-GFP vector (addgene Plasmid #16578) between BamH1 cloning sites for transiently expressing the genes in Retinal pigment epithelial cells (RPE1) cells. For inducible expression of the genes, the CPAP-GFP constructs were cloned into PLIX backbone using gateway cloning (PLIX403 was a gift from David Root- addgene plasmid 41395). RNAi-resistant CPAP BACs were prepared and described as previously described [1, 6].

Lentiviral production

For carrying out lentiviral transductions, the GFP tagged $CPAP^{WT}$, $CPAP^{F375A}$ and $CPAP^{EE343RR}$ were cloned into the pSinEF-2-GFP vector. For packaging the virus, pMD2, pRSV and pMDL plasmids were used. Further, HEK293T cells were transfected with the packaged products along with the pLenti plasmids using calcium chloride. After 24 hrs of transfection, the first batch of virus was collected followed by the second batch that was collected after 36 hrs.

Cell Culture, transfection and viral transduction

Wild type RPE1 and HeLa cells were cultured in a medium containing Dulbecco's modified Eagle's medium (DMEM), 10% (v/v) fetal bovine serum (FBS), 0.1 mM MEM non-essential amino acids (NAA), 100 µg ml^{-1} streptomycin, 100 U ml^{-1} penicillin (from life Technologies GmbH, Dermstadt, Germany). The cells were incubated at 37^0C and split after they reached a confluency of 70-80%. For induction of cilium in RPE1, the cells were serum starved for different time periods by growing them in a medium that lacks serum. For cilium resorption experiments, 10 % FBS was added to the serum starved medium and incubated for 24 to 48 hrs. RPE1 cells were seeded on coverslips and transduced with lentiviral particles (1:10 dilution), harboring GFP tagged $CPAP^{WT}$, $CPAP^{F375A}$ and $CPAP^{EE343RR}$ genes. The transduced cells were incubated for a minimum of 48 hrs before further treatment. For the inducible lentiviral system, 2µg ml^{-1} of doxycycline was added to the cultures to induce the expression of CPAP. The cells were incubated for 2 hrs before taking measurements to

ensure sufficient protein expression. For RNAi experiments, the siRNA-resistant versions of CPAP with the above mentioned point mutations were generated as described previously [6].

Immunofluorescence and light microscopy

For immunofluorescence, mouse anti-CPAP (1:25) *(2),* rat anti-α-tubulin (1:50 chemicon), rabbit anti-Cep 152 (1:500 provided by the Nigg laboratory), rabbit anti-Arl13b (1:100, Proteintech, Manchester, UK), mouse anti-acetylated tubulin (1:500, Sigma-Aldrich), rabbit anti-CP110 (1:500 Proteintech), rabbit anti-cep 164 (1:1000 provided by Nigg laboratory), rabbit anti-Asl (1:5000) were used. Secondary antibodies, Alexa fluor dyes (goat or donkey anti-mouse/ anti-rabbit) were used at 1:1000 dilution (Life technologies). DAPI (1 μg ml^{-1}. Sigma) was used to stain DNA.

For light microscopy, the required amount of cells were seeded on coverslips and allowed to adhere. After experimental treatment, the cells were either fixed using 3.7% para-formaldehyde (PFA) or ice-cold methanol. After fixation, the specimens were permeabilized with 0.5% triton X-100 in PBS for 10 mins. 0.5% fish gelatin in PBS was used to block the permeabilized samples at room temperature for 2 hrs. Primary antibody labeling was carried out either at RT for 1 hr or overnight at 4ºC. After labeling, the antibody was removed and the samples were washed thrice with PBS. Further, the respective secondary antibodies along with DAPI were used against the primary antibody and incubated at room temperature for 1 hr. Confocal images were obtained using an Olympus Fluoview FV 1000 scanning confocal microscope. The images were further processed using Image J and Adobe Photoshop. For the live cell imaging of RPE1, the cells were grown on ibidi microscopic chamber (Cat.No.80296) was performed with Leica DMI 6000B using 40X water objective and 63X oil objective.

For light microscopy of fly testes, the dark pupae testes were dissected in PBS and permeabilized with 0.1% Triton X-100 in PBS for 10 mins. The samples were blocked with 2 % BSA, 0.1% tritonX-100 in PBS for 1 hr at room temperature. The samples were labeled with primary antibodies at RT for 1-2hr or overnight at 4ºC. The samples were further washed and labeled with secondary antibodies for 1 hr at RT along with DAPI. Analyses of the samples were carried out as described earlier.

Electron microscopy

For EM of RPE1 cells stably expressing the CPAP mutants, the cells were seeded onto

coverslips and allowed to adhere. The cells were further serum starved for 48 hrs to induce cilium formation. 2% glutaraldehyde (Electron Microscopy Sciences) was used to fix the cells. The fixed cells were further processed and then used for electron microscopy as described earlier [6].

For EM of drosophila testes, the testes from the different Sas-4 transgenic flies were dissected and fixed using 2% glutaraldehyde. The fixed samples were then processed and used for electron microscopy. The images obtained were processed using Adobe photoshop.

Western Blotting

Protein samples in 4x laemlli buffer were heated at 98ºC for 10 mins before use. The samples were resolved using 8% acrylamide gels and transferred to nitrocellulose membranes. The blots were further blocked with 5% non-fat dried milk powder in TBST for 1 hr and incubated overnight at 4ºC with the primary antibodies. The blots were later treated with the respective secondary antibodies at RT for 1 hr. Super signal west pico/ femto chemilluminescent substrates were used to detect the peroxidase activity in the samples.

Generation of transgenic flies

Genomic Sas-4^{WT} (control)**,** Sas-4^{F112A} and Sas-4^{EE78RR} were cloned into PattB-GFP between AscI and NotI sites. The plasmids were injected into the larvae as described [7]. All the genes were expressed under Sas-4 endogenous promoter and were inserted at the identical sites at the third chromosome using PattB vectors. The flies were balanced to generate a stable stock. For rescue experiments, Sas-4^{WT}, Sas-4^{F112A} and Sas-4^{EE78RR} were expressed in the background of Sas-4 null homozygous flies, and analyzed for their fly co-ordination, centriole and cilium length.

Statistical Analysis

Statistical analysis was carried out using Graph Pad Prism software (version 6.0). Student's T- test or one-way ANOVA followed by Tukey's multiple comparison tests were used to analyze the results.

Supplementary References

1. Zheng, X. *et al.* Conserved TCP domain of Sas-4/CPAP is essential for pericentriolar material tethering during centrosome biogenesis. *Proceedings of the National Academy of Sciences of the United States of America* **111**, E354-363 (2014).
2. Schmidt, T.I. *et al.* Control of centriole length by CPAP and CP110. *Current biology : CB* **19**, 1005-1011 (2009).
3. Kobayashi, T., Tsang, W.Y., Li, J., Lane, W. & Dynlacht, B.D. Centriolar kinesin Kif24 interacts with CP110 to remodel microtubules and regulate ciliogenesis. *Cell* **145**, 914-925 (2011).
4. Lin, Y.N. *et al.* CEP120 interacts with CPAP and positively regulates centriole elongation. *The Journal of cell biology* **202**, 211-219 (2013).
5. Bieling, P., Telley, I.A., Hentrich, C., Piehler, J. & Surrey, T. Fluorescence microscopy assays on chemically functionalized surfaces for quantitative imaging of microtubule, motor, and +TIP dynamics. *Methods in cell biology* **95**, 555-580 (2010).
6. Poser, I. *et al.* BAC TransgeneOmics: a high-throughput method for exploration of protein function in mammals. *Nature methods* **5**, 409-415 (2008).
7. Fish, M.P., Groth, A.C., Calos, M.P. & Nusse, R. Creating transgenic Drosophila by microinjecting the site-specific phiC31 integrase mRNA and a transgene-containing donor plasmid. *Nature protocols* **2**, 2325-2331 (2007).
